# Analysis of Radiocarbon, Stable Isotopes and DNA in Teeth to Facilitate Identification of Unknown Decedents

**DOI:** 10.1371/journal.pone.0069597

**Published:** 2013-07-29

**Authors:** Kanar Alkass, Hisako Saitoh, Bruce A. Buchholz, Samuel Bernard, Gunilla Holmlund, David R. Senn, Kirsty L. Spalding, Henrik Druid

**Affiliations:** 1 Division of Forensic Medicine, Department of Oncology-Pathology, Karolinska Institutet, Stockholm, Sweden; 2 Department of Legal Medicine, Graduate School of Medicine, Chiba University, Chiba, Japan; 3 Center for Accelerator Mass Spectrometry, Lawrence Livermore National Laboratory, Livermore, California, United States of America; 4 Institut Camille Jordan, CNRS UMR 5208, University of Lyon, Villeurbanne, France; 5 Department of Forensic Genetics and Forensic Toxicology, National Board of Forensic Medicine, Linköping, Sweden; 6 Center for Education and Research in Forensics, The University of Texas Health Science Center at San Antonio, San Antonio, Texas, United States of America; 7 Department of Cell and Molecular Biology, Karolinska Institutet, Stockholm, Sweden; University of Illinois at Champaign-Urbana, United States of America

## Abstract

The characterization of unidentified bodies or suspected human remains is a frequent and important task for forensic investigators. However, any identification method requires clues to the person’s identity to allow for comparisons with missing persons. If such clues are lacking, information about the year of birth, sex and geographic origin of the victim, is particularly helpful to aid in the identification casework and limit the search for possible matches. We present here results of stable isotope analysis of ^13^C and ^18^O, and bomb-pulse ^14^C analyses that can help in the casework. The ^14^C analysis of enamel provided information of the year of birth with an average absolute error of 1.8±1.3 years. We also found that analysis of enamel and root from the same tooth can be used to determine if the ^14^C values match the rising or falling part of the bomb-curve. Enamel laydown times can be used to estimate the date of birth of individuals, but here we show that this detour is unnecessary when using a large set of crude ^14^C data of tooth enamel as a reference. The levels of ^13^C in tooth enamel were higher in North America than in teeth from Europe and Asia, and Mexican teeth showed even higher levels than those from USA. DNA analysis was performed on 28 teeth, and provided individual-specific profiles in most cases and sex determination in all cases. In conclusion, these analyses can dramatically limit the number of possible matches and hence facilitate person identification work.

## Introduction

The identification of unknown bodies is crucial for ethical, medico-legal and civil reasons. Police and forensic investigators worldwide spend considerable time every year attempting to solve such cases. Typically, unidentified bodies or human remains are eventually identified by fingerprint comparisons, comparison of ante- and postmortem dental or medical radiographs, or by DNA analysis of tissue from the dead body and from possible relatives. However, in cases where there are no clues as to the identity, a characterization of the body can limit and focus the search for possible matches and help to exclude impossible or unlikely alternatives. The Doe Network Database (http://www.doenetwork.org) is extensively used in North America and includes both reported missing persons and unidentified bodies. This database contains information on about 3,800 unidentified deceased individuals, the majority from the United States. A careful review of this database (Table 1) showed that the estimated age for adult individuals displayed an average uncertainty range of 15±12 years. Such a wide range is not very helpful in the efforts to limit the search for possible matches. Furthermore, the sex was unknown or uncertain in some of the cases, and the geographic origin of the person rarely known, apart from information about the place where the victim was found. Hence, a more detailed characterization of the subjects in terms of age, sex and geographical origin is expected to improve the success rate in dead victim identification work.

**Table 1:** Panorama of unidentified bodies registered in the Doe Network database April 2011.

No. of Cases	Male (%)	Asian (%)	Black (%)	Hisp (%)	White (%)	N/A (%)	Avg. low^1^	Avg. high^1^	Error range^2^	>1 yr 3 (%)
1229	66.7	2.6	51.7	6.2	20.6	18.9	29.1	43.0	15.0	49.0

1 The average minimum and maximum estimated age of the dead body.

2 The average possible age range for all cases, where such estimation is provided.

3 This figure represents the number of cases that still are unidentified after more than a year.

The most common procedure to estimate the age of an unidentified body is to perform an anthropological examination, which regarding skeletonized bodies focuses on age-related changes in teeth and bones. However, anthropological methods are not very precise and typically an error of ±10 years is added to the estimated age [1]. Additional methods have therefore been developed. Today, the most precise method to determine the age at death is aspartic acid racemization analysis, which is based on the observation that remaining aspartic acid in the tooth trapped during its formation will be converted at a very slow rate from the L-form to the D-form, both of which can be detected and quantified by gas chromatographic methods [2]. These methods applied on enamel, dentin or cementum can provide an estimate of the age of a person with a fairly good precision; using dentin the error may be ±5 years or less [3], [4], [5]. All the methods used to determine age are based on age-dependent alterations in human tissues, and hence they will all give an estimate of the person’s age at death. In contrast, the recently developed method to analyze bomb-pulse ^14^C in enamel indicates the year of birth regardless of when, or at what age, the person died [6]. This method should not be confused with the Libby method [7] to date archeological material, which is based on the radioactive decay of ^14^C in biological material. Instead, the bomb-pulse ^14^C method takes advantage of the substantial increase in global ^14^C levels caused by above-ground nuclear test bomb detonations 1955–1963 [8.9.10,11,12]. Repeated measurements of ^14^C in the atmosphere and in biological products of known age, has over time resulted in reference values to which analytical results can be compared to offer an estimate of the age; see http://calib.qub.ac.uk/CALIBomb [10].

Similarly, an important factor for limiting the search for possible matches is the sex of the individual. Unknown human bodies that are fairly well preserved rarely pose any problem, but if the forensic case involves a mutilated or skeletonized body, anthropological examination of bones that show sex dimorphism is often performed. DNA profiling of bones and teeth is possible, and can include markers for sex. However, if a fire victim is charred, extraction and amplification of DNA from soft tissues and bones can be difficult. Teeth represent the most resistant tissue in the body and DNA analysis of teeth may be an alternative. Such analysis, including markers for sex, has successfully been performed even on incinerated bodies [13].

To limit the search for possible matches, the geographic origin of the deceased may also provide clues to the identity. In birds, analysis of stable isotopes in feathers formed during the winter has been used to determine wintering areas [14]. In a recent study, hair samples from subjects living on different continents displayed geographically specific isotopic signatures [15]. In a previous study, we have shown that such geographic differences regarding ^13^C also can be detected in teeth [16]. This isotope is incorporated into the tissues of animals, including humans, in relation to the content in food. Since the diet by tradition varies geographically, and is based on different primary products derived from plants with different ^13^C content, the amounts in tissues will vary accordingly [17]. The geographic differences can be explained as follows. Some types of plants can discriminate between ^12^C and ^13^C, resulting in differences in the levels of ^13^C between different types of plants. C4 plants (which include corn and sugar cane) contain higher amounts of ^13^C than C3 plants (which include potato, wheat and sugar beet) since these strive to maximize their CO_2_ assimilation. In general, C4 plants tend to grow in hotter or drier climates than C3 plants. This in turn means that animals, including humans, having a diet based mainly of C4 plants and/or on animals that eat C4 plants, will incorporate more ^13^C than those mainly living off C3 plant based diets.

^18^O is another stable isotope that shows geographic variation. The incorporation of ^18^O in animal tissues is correlated to the levels in drinking water and these levels vary with latitude because of differences in the evaporation and condensation propensity between ^16^O and ^18^O [18]. In the United States, reports indicate that the highest tap water ^18^O concentrations are in the south and southeast and the lowest in the northwest [19]. These data are based on tap water samples collected from 2002–2003. Due to differences in precipitation and variable ground water supplies to specific areas, these levels may differ somewhat from year to year. Still, ^18^O levels are not expected to change dramatically over time and hence levels in teeth, bones and hair should mirror the levels in tap water fairly well.

In this study we investigated whether analysis of bomb-pulse derived ^14^C levels in teeth from subjects in North America (Table 2) shows similar precision as in previous studies on teeth from other continents. In addition, we analyzed ^14^C in both enamel and roots of the same teeth to find out if this measure could help to discriminate values that relate to incorporation during the rising or falling part of the bomb-curve (Table 3). We also wanted to explore possible geographical variation in ^13^C (Table 4) and ^18^O (Table 5) by collecting teeth from Mexico, Canada and different parts of the United States. From the same teeth, we also performed DNA analysis of the amelogenin gene to determine the sex of the subjects. Finally, the average enamel ^14^C incorporation time in different types of teeth from this study were combined with the results from previous studies [5], [6], [16] in order to provide a reference guide for determining the date of birth of persons (Table 6).

**Table 2:** Overall results of ^14^C dating of teeth collected in North America.

					Radiocarbon analysis		Teeth		Person			
CaseNo.	Sex	ToothNo.	Enamelformationtime (yrs)^1^	Raisedin/collectedin	Fractionmodern	±	ActualtoothDOB^1^	Estimatedtooth DOB^2^	ActualDOB	EstimatedDOB	Error	Absoluteerror
1	M	41	2.5	BC	1.5410	0.0067	1965.3	1963.1	1962.8	1960.6	−2.2	2.2
2	F	42	2.8	BC	0.9986	0.0038	1918.4	Pre-bomb	1915.6	Pre-bomb	Pre-bomb	Pre-bomb
3	M	15	6.6	BC	1.5361	0.0045	1974.0	1970.1	1967.4	1963.5	−3.9	3.9
4	M	44	5.1	BC	1.1130	0.0042	2001.7	1996,3	1996.6	1991.2	−5.4	5.4
5	F	36	2.3	BC	1.3645	0.0051	1972.6	1967.4	1970.4	1974.2	3.8	3.8
6	M	22	4.0	BC	1.6698	0.0072	1966.2	1966.8	1962.2	1962.9	0.7	0.7
7	M	38	13.0	WA	1.1357	0.0040	1995.3	1992.5	1982.3	1979.5	−2.8	2.8
	M	48	13.0	WA	1.1323	0.0042	1995.3	1993.1	1982.3	1980.1	−2.2	2.2
8	F	27	5.8	WA	1.6459	0.0059	1968.6	1967.2	1962.8	1961.4	−1.4	1.4
	F	45	5.7	WA	1.6356	0.0057	1968.5	1967.4	1962.8	1961.7	−1.1	1.1
9	M	17	6.5	WA	1.1820	0.0042	1989.8	1987.5	1983.3	1981.0	−2.3	2.3
10	M	47	6.5	WA	1.3421	0.0047	1979.9	1977.5	1973.4	1971.0	−2.4	2.4
11	F	24	4.9	WA	1.3585	0.0050	1964.9	1962.4	1960.0	1957.5	−2.5	2.5
12	M	16	3.3	WA	1.4898	0.0053	1973.9	1971.8	1970.6	1968.5	−2.1	2.1
	M	37	6.5	WA	1.3518	0.0048	1977.1	1976.8	1970.6	1970.3	−0.3	0.3
13	M	15	6.6	WA	1.1170	0.0040	1998.4	1995.6	1991.8	1989.0	−2.8	2.8
14	M	17	6.5	MT/WA	1.1646	0.0041	1991.0	1989.4	1984.5	1982.9	−1.6	1.6
	M	18	12.6	MT/WA	1.1242	0.0060	1997.1	1994.3	1984.5	1981.7	−2.8	2.8
	M	28	12.6	MT/WA	1.1118	0.0039	1997.1	1996.5	1984.5	1983.9	−0.6	0.6
15	F	25	5.6	MT/WA	1.3675	0.0048	1975.4	1975.8	1969.8	1970.2	0.4	0.4
	F	26	3.0	MT/WA	1.4750	0.0052	1972.8	1972.2	1969.8	1969.2	−0.6	0.6
16	F	26	3.0	UK/WA^3^	1.1765	0.0046	1990.6	1988.0	1987.6	1985.0	−2.6	2.6
17	M	45	6.5	MA	1.2773	0.0036	1964.2	1962.0	1957.7	1956.0	−1.7	1.7
18	M	44	5.1	CT	1.0940	0.0039	2002.3	2000.0	1997.2	1994.9	−2.3	2.3
	M	14	5.6	CT	1.0922	0.0031	2002.8	2000.0	1997.2	1994.4	−2.8	2.8
19	M	14	5.6	CT	1.0971	0.0038	2001.9	1999.2	1996.3	1993.6	−2.7	2.7
	M	34	5.1	CT	1.0872	0.0038	2001.4	2001.0	1996.3	1995.9	−0.4	0.4
20	F	31	2.5	CA	1.0134	0.0036	1947.4	Pre-bomb	1944.9	Pre-bomb	Pre-bomb	Pre-bomb
	F	33	4.1	CA	0.9920	0.0029	1949.0	Pre-bomb	1944.9	Pre-bomb	Pre-bomb	Pre-bomb
21	M	15	6.6	CA	1.0496	0.0044	1959.7	1957.3	1953.1	1950.7	−2.4	2.4
22	M	28	12.6	CA/WA	1.1462	0.0040	1992.8	1991.6	1980.2	1979.0	−1.2	1.2
23	M	42	3.0	GA	1.2281	0.0038	1984.1	1983.3	1981.1	1980.3	−0.7	0.7
24	F	23	4.7	AL	1.0171	0.0036	1958.1	1956.1	1953.4	1951.4	−2.0	2.0
25	F	14	4.9	TX	1.1396	0.0040	1996.3	1992.1	1991.4	1987.2	−4.2	4.2
	F	34	4.4	TX	1.1224	0.0032	1995.8	1994.6	1991.4	1990.2	−1.2	1.2
26	M	14	5.6	TX	0.9948	0.0028	1945.9	Pre-bomb	1940.3	Pre-bomb	Pre-bomb	Pre-bomb
	M	31	2.5	TX	0.9900	0.0032	1942.8	Pre-bomb	1940.3	Pre-bomb	Pre-bomb	Pre-bomb
27	M	23	4.7	TX	1.1726	0.0036	1960.8	1958,8	1956.1	1954.1	−2.0	2.0
28	M	36	2.4	TX	1.1019	0.0035	1994.4	1998.2	1992.0	1995.8	3.8	3.8
29	M	18	12.6	TX	1.3799	0.0038	1976.5	1975.4	1965.3	1962.8	−2.5	2.5
	M	46	2.4	TX	1.4075	0.0041	1967.6	1962.7	1965.3	1960.3	−5.0	5.0
30	M	12	4.0	TX	1.3087	0.0038	1964.1	1962.1	1960.1	1958.1	−2.0	2.0
	M	13	4.7	TX	1.3502	0.0041	1964.8	1962.4	1960.1	1957.7	−2.4	2.4
	M	14	5.6	TX	1.4744	0.0062	1965.7	1962.9	1960.1	1957.3	−2.8	2.8
	M	22	4.0	TX	1.6172	0.0066	1964.1	1963.3	1960.1	1959.3	−0.8	0.8
	M	23	4.7	TX	1.7399	0.0267	1964.8	1965.8	1960.1	1961.1	1.0	1.0
	M	26	3.3	TX	1.2254	0.0046	1963.4	1961.1	1960.1	1957.8	−2.3	2.3
	M	31	2.5	TX	1.2791	0.0073	1962.6	1962,0	1960.1	1959.5	−0.6	0.6
	M	32	3.0	TX	1.6571	0.1693	1963.1	1963.3	1960.1	1960.3	0.2	0.2
	M	37	6.5	TX	1.6735	0.0056	1966.6	1966.8	1960.1	1960.3	0.2	0.2
	M	41	2.5	TX	1.4131	0.0059	1962.6	1962.6	1960.1	1960.1	0.0	0.0
	M	42	3.0	TX	1.5051	0.1504	1963.1	1962.8	1960.1	1959.8	−0.3	0.3
	M	43	4.3	TX	1.4475	0.0062	1964.4	1962.8	1960.1	1958.5	−1.6	1.6
	M	44	5.1	TX	1.6666	0.0067	1965.2	1966.4	1960.1	1961.3	1.2	1.2
	M	47	6.5	TX	1.6945	0.0060	1966.6	1966.5	1960.1	1960.0	−0.1	0.1
31	M	11	3.2	TX	1.3500	0.0048	1979.6	1977.0	1976.4	1973.8	−2.6	2.6
32	F	46	2.4	Mexico	0.9849	0.0035	1946.9	Pre-bomb	1944.5	Pre-bomb	Pre-bomb	Pre-bomb
33	M	23	4.7	Mexico	1.0280	0.0040	1956.8	1956.0	1952.1	1951.3	−0.8	0.8
34	M	26	3.3	Mexico	1.2163	0.0045	1962.8	1960.0	1959.5	1956.7	−2.8	2.8
35	F	46	2.3	Mexico	0.9945	0.0035	1945.7	Pre-bomb	1943.4	Pre-bomb	Pre-bomb	Pre-bomb
36	M	37	6.5	Mexico	1.6573	0.0059	1961.3	1962.7	1954.8	1956.2	1.4	1.4
	M	36	2.4	Mexico	1.0738	0.0038	1957.2	1957.4	1954.8	1955.0	0.2	0.2
37	F	35	5.7	Mexico	0.9920	0.0033	1926.7	Pre-bomb	1921.0	Pre-bomb	Pre-bomb	Pre-bomb
38	F	36	2.3	Mexico	0.9949	0.0034	1933.5	Pre-bomb	1931.2	Pre-bomb	Pre-bomb	Pre-bomb
39	F	17	5.8	Mexico	1.5306	0.0054	1969.6	1970.1	1963.8	1964.3	0.5	0.5
	F	45	5.7	Mexico	1.5331	0.0058	1969.5	1970.2	1963.8	1964.5	0.7	0.7
											**Average**	**1.8**
											**SD**	**1.3**

1 Enamel formation time according to Nolla [20].

2 Average age of carbonate in dental enamel based on ^14^C measurement.

3 Unclear at what age this subject moved to WA.

**Table 3:** ^14^C levels in enamel and roots from the same teeth.

			Enamel		Root	
			Radiocarbon analysis			
CaseNo.	ToothNo.	Actual enamelDOB	FractionModern	±	Fractionmodern	±
2	42	1918.4	0.9986	0.0038	1.0579	0.0037
35	46	1945.7	0.9945	0.0035	1.0586	0.0037
26	14	1945.9	0.9948	0.0028	1.0194	0.0036
32	46	1946.8	0.9849	0.0035	1.0794	0.0038
20	33	1949.0	0.9920	0.0029	1.0662	0.0038
33	23	1956.8	1.0280	0.0040	1.4106	0.0050
24	23	1957.2	1.0171	0.0036	1.3740	0.0048
21	15	1959.7	1.0496	0.0044	1.2139	0.0048
27	23	1960.8	1.1726	0.0036	1.4541	0.0051
36	37	1961.3	1.6573	0.0059	1.4043	0.0056
30	32	1963.1	1.6571	0.1693	1.4007	0.0051
30	42	1963.1	1.5051	0.1504	1.4084	0.0051
1	41	1965.3	1.5410	0.0067	1.4372	0.0051
30	37	1966.6	1.6735	0.0056	1.3785	0.0044
39	45	1969.5	1.5331	0.0058	1.2894	0.0047
3	15	1974	1.5361	0.0045	1.2656	0.0044
15	25	1975.4	1.3675	0.0048	1.1948	0.0047
29	18	1977.9	1.3799	0.0038	1.1660	0.0042
31	11	1979.6	1.3500	0.0048	1.1708	0.0039
9	17	1989.8	1.1820	0.0042	1.1127	0.0037
16	26	1990.6	1.1765	0.0046	1.0910	0.0038
14	17	1991	1.1646	0.0041	1.0891	0.0043
22	28	1992.8	1.1462	0.0040	1.0865	0.0038
25	14	1996.3	1.1396	0.0040	1.0737	0.0038
4	44	2001.7	1.1130	0.0042	1.0575	0.0037
19	14	2001.9	1.0971	0.0038	1.0478	0.0039
18	14	2002.8	1.0922	0.0031	1.0442	0.0037
28	18	2004.6	1.1158	0.0032	1.0867	0.0049

**Table 4:** δ^13^C levels in teeth from individuals raised in different parts of North America.

Case No.	Sex	Tooth No	Enamel formationtime (yrs)^1^	Raised in	δ^13^C enamel	δ^13^C roots
3	M	15	6.6	BC	−11.78	−13.40
4	M	44	5.1	BC	−11.48	−12.22
5	F	36	2.3	BC	−10.99	
6	M	22	4	BC	−10.00	
12	M	37	6.5	WA	−11.74	
12	M	16	3.3	WA	−11.50	
11	F	24	4.9	WA	−10.35	
13	M	15	6.6	WA	−10.33	
9	M	17	6.5	WA	−10.03	−12.31
7	M	48	13	WA	−9.45	
7	M	38	13	WA	−9.38	
14	M	17	6.5	MT	−11.27	−6.27
15	F	26	3	MT	−11.07	
14	M	28	12.6	MT	−11.03	
17	M	45	6.5	MA	−7.89	
19	M	14	5.6	CT	−10.84	−12.20
18	M	14	5.6	CT	−10.82	−10.41
19	M	34	5.1	CT	−10.56	
22	M	28	12.6	CA	−12.56	−12.28
21	M	15	6.6	CA	−10.51	−11.28
20	F	33	4.1	CA	−10.39	−11.95
23	M	42	3	GA	−8.98	
24	F	23	3.8	AL	−9.02	−10.91
28	M	36	2.4	TX	−10.19	
25	F	14	4.9	TX	−9.54	−10.97
30	M	26	3.3	TX	−9.20	
25	F	34	4.4	TX	−9.18	
30	M	12	4.0	TX	−9.04	
29	M	46	2.4	TX	−8.96	−10.65
29	M	18	12.6	TX	−8.85	
26	M	14	5.6	TX	−8.83	−10.17
27	M	23	4.7	TX	−8.41	−9.92
36	M	37	6.5	Mexico	−7.78	−6.32
39	F	45	5.7	Mexico	−6.37	−9.55
36	M	36	2.4	Mexico	−6.29	
33	M	23	4.7	Mexico	−6.23	−8.77
34	M	26	3.3	Mexico	−5.76	
35	F	46	2.3	Mexico	−5.71	−6.80
32	F	46	2.3	Mexico	−4.32	−10.60
37	F	35	5.7	Mexico	−0.54	
38	F	36	2.3	Mexico	0.11	

1 Enamel formation time according to Nolla [20].

**Table 5:** δ^ 18^O levels in tooth roots.

Case No.	Tooth No.	Raised in/collected in	d^18^O Root
3	15	BC	−8.03
1	41	BC	−6.23
2	42	BC	−9.88
4	44	BC	−8.12
9	17	WA	−7.14
16	26	UK/WA^1^	−6.35
15	25	MT/WA	−8.17
14	17	MT/WA	−5.67
19	14	CT	−6.26
18	14	CT	−5.00
22	28	CA/WA	−7.87
20	33	CA	−5.61
22	18	CA/WA	−8.12
21	15	CA	−4.81
24	23	AL	−8.15
30	42	TX	−9.01
31	11	TX	−5.48
28	18	TX	−5.15
25	14	TX	−5.14
30	32	TX	−5.11
29	18	TX	−5.64
30	37	TX	−5.16
26	14	TX	−5.02
27	23	TX	−5.95
30	33	TX	−5.44
32	46	Mexico	−7.16
39	45	Mexico	−6.60
33	23	Mexico	−6.43
37	37	Mexico	−6.38
35	46	Mexico	−6.24
36	37	Mexico	−6.43

1 Unclear at what age this subject moved to WA.

**Table 6:** Reference information for calculating the date of birth of the person using enamel laydown time and ^14^C incorporation time.

Tooth No.	Enamelformation time^1^	^14^C incorporationtime^2^	Absolute average errorusing enamel formation time^1^	Absolute average errorusing ^14^C incorporation time^2^
11/21	3.2	2.2±1.4	1.5±0.8	1.0±0.9
12/22	3.9	3.8±1.0	0.8±0.6	0.9±0.5
13/23	4.4	3.3±1.3	1.5±0.8	1.1±0.7
14/24	5.2	3.6±1.2	1.7±1.1	1.0±0.7
15/25	6.2	4.8±1.3	1.5±1.1	1.1±0.7
16/26	3.2	1.5±1.2	1.8±1.0	1.0±0.5
17/27	6.1	5.1±1.0	1.2±0.6	0.8±0.6
18/28	12.1	11.5±1.9	1.6±1.1	1.4±1.2
31/41	2.5	1.9±0.8	0.6±0.7	0.6±0.5
32/42	3.0	3.2±2.5	1.4±1.9	1.5±1.8
33/43	4.3	3.6±0.9	0.8±0.7	0.7±0.2
34/44	4.9	4.1±1.7	1.4±1.2	1.1±1.2
35/45	6.0	5.3±0.8	0.8±0.7	0.7±0.3
36/46	2.4	3.0±3.0	2.4±1.7	2.2±1.9
37/47	6.2	6.2±1.6	1.2±0.9	1.1±1.1
38/48	12.6	11.0±1.9	1.5±1.1	1.1±1.0
		**Total.**	**1.4±0.4**	**1.1±0.5**

1 Enamel formation time according to Nolla [20].

2 The time interval used for error calculation does not include lag of ^14^C in the food chain.

## Results

### Date of Birth (DOB) Estimation Using Tooth Enamel

For all 66 teeth, sufficient amounts of enamel were obtained to allow for ^14^C determination by accelerator mass spectrometry (AMS). Table 2 displays the overall results and Table S1 provides more detailed information. Nine of the teeth had a calculated enamel formation time before 1955 and all showed pre-bomb ^14^C values. For teeth laid down after 1955, a high correlation was found between ^14^C levels in enamel and the actual formation time of the enamel, with an average absolute error of 1.8±1.3 years, R^2^ = .9935 (n = 57). The error of the estimates obtained using Nolla [20] and crude ^14^C, respectively are given in Table S2, and is further described in detail in Supporting online information. Table S3 in Supporting Information S1 shows a comparison between the Nollás calibration and crude ^14^C measurement in age estimation. Table S4 in Supporting Information S1 provides standard deviation in age at tooth formation for each tooth type and jaw. The confidence intervals for the enamel DOB based on ^14^C incorporation time are given in Table S5 in Supporting Information S1. In 14 cases, two or more teeth were analyzed from the same individual. On average the mean difference in estimates of date of birth of the person between teeth from the same individuals was 1.4 (median 1.2) years. In Case 30, 14 teeth had been extracted. The ^14^C values of each of these teeth predicted the true DOB very well and the difference in prediction of DOB between these teeth was small (Table 2). In nine of the twelve cases, where the enamel was formed after the onset of the bomb pulse, enamel ^14^C values matched the expected order of formation, i.e. teeth with later formation time showed more recent ^14^C levels. In Cases 8 and 19 both teeth from each individual showed very similar ^14^C values since both tooth types had similar enamel formation times (Table 2). In Case 30, high ^14^C values were seen in all teeth, which was in line with the fact that their actual formation dates were during the peak of the bomb curve.

### ^14^C Incorporation in Tooth Roots

We also analyzed ^14^C levels in the roots from 28 teeth collected from 26 individuals (Table 3). The ^14^C levels were consistently higher in the roots than in the enamel if the enamel was formed during the rising part of the curve, and lower in the roots if the enamel was formed during the falling part of the curve. One exception was Case 36, where the enamel was formed shortly before the peak of the bomb-spike and most likely the lower level of ^14^C in the root is explained by lower levels incorporated during the falling part of the curve. In all pre-bomb cases, i.e. when the enamel was completely formed before the bomb pulse, post-bomb values were found in their corresponding roots, indicating that a turnover in some component of the root continues even into old age, as evidenced by the elevated ^14^C concentration in a man born in 1918 (Case 2). This finding confirms previous observations of a continuous formation of secondary dentin and cementum throughout life [21], [22].

### Reference Levels of ^14^C to Determine Date of Birth

**Figure 1:**
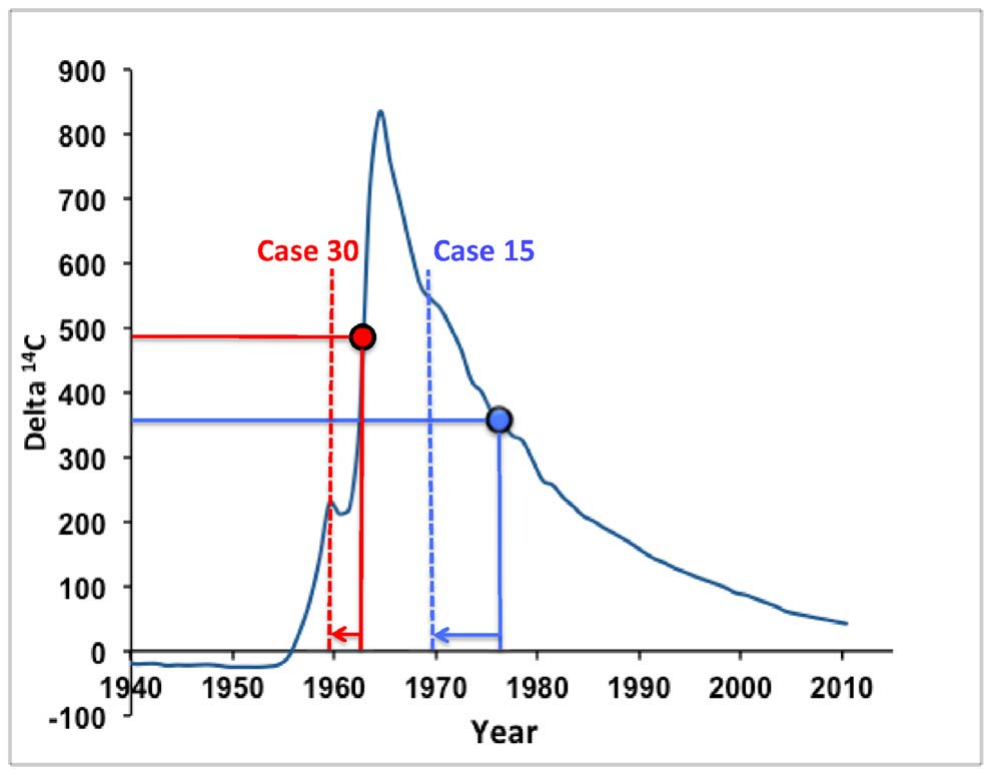
The principle for age estimation by analyzing ^14^C in dental enamel, exemplified by two teeth from cases 30 and 15, respectively. Horizontal lines represent their radiocarbon values crossing the ^14^C bomb curve, and corresponding date of enamel formation indicated by the vertical lines crossing the abscissa. By subtracting the average formation time (arrows) for each tooth, the DOB of the person can be calculated (dashed vertical lines). The true DOB was 0.3 and 0.4 years, respectively, from the estimated DOB of these persons.

The principle for DOB determination of persons using ^14^C has been based on subtracting the enamel formation time for the particular type of tooth based on radiographic mapping of tooth development (Fig. 1). However, in Table S2, we have compiled the enamel DOB as estimated by ^14^C levels in this and previous studies [5], [6], [16] and compared the result for each type of tooth with the actual DOB of the person. We show that the error in birth dating can be reduced by subtracting the average enamel ^14^C incorporation time instead.

### ^13^C in Teeth can Tell Geographic Origin

^13^C levels in the tooth enamel from 41 teeth (33 individuals) were analyzed with isotopic ratio mass spectrometry (Table 4). The teeth extracted from subjects raised in North America showed higher enamel ^13^C values compared to levels previously seen in teeth collected from other continents, including South America [16]. The levels were somewhat lower in teeth from California, British Columbia and Connecticut, compared to teeth from Texas. Enamel from teeth from Mexico showed very high levels, even higher than levels in enamel from teeth from the United States and Canada, without overlap. ^13^C levels in 31 tooth roots from 27 individuals were also analyzed; 19 of the roots were from the same teeth in which ^13^C enamel levels also were measured (Table 4). The ^13^C levels in the roots were generally lower than in the enamel. Furthermore, the ^13^C levels in tooth roots were higher in teeth from Mexico than in roots from the United States and Canada, but showed a higher variation, making it more difficult to separate Mexican subjects from persons raised in United States and Canada using roots only.

### Tap Water Provides an Additional Signature

**Figure 2:**
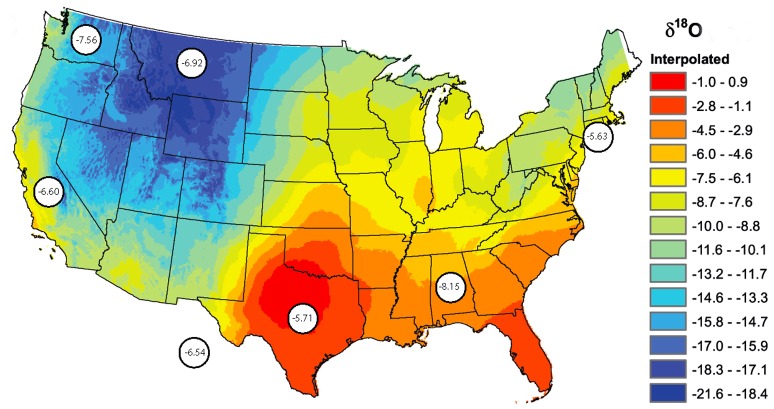
Map of ^18^O in drinking water across the United States. The encircled values represent the average levels in tooth roots from subjects raised in the different states. Values from subjects from British Columbia and Washington are merged. The map is reproduced from [19] with permission by the publisher (American Geophysical Union, www.agu.org).

In Table 5, the ^18^O levels in all roots analyzed are displayed. ^18^O levels were in general lower in tooth roots from the northwestern region of North America (Washington and British Columbia) compared to those in roots collected from Texas (p<0.01). One subject from Alabama and one subject from Texas were outliers, but otherwise the levels of ^18^O paralleled the tap water levels fairly well (Fig. 2). Mexican teeth showed slightly lower values than teeth from Texas and were similar to the average for all teeth from the United States and Canada. From Table 5, it can be appreciated that there are regional differences in the tooth levels of ^18^O, but at a lower magnitude compared to the difference in tap water levels. Since we did not collect information about the exact place where the subjects were raised, interstate differences may explain the modest differences between states. Furthermore, levels of ^18^O are also dependent on diet composition, since a large part of the oxygen incorporated in tissues derives from food that may be produced at remote locations.

### Sex Determination

**Figure 3:**
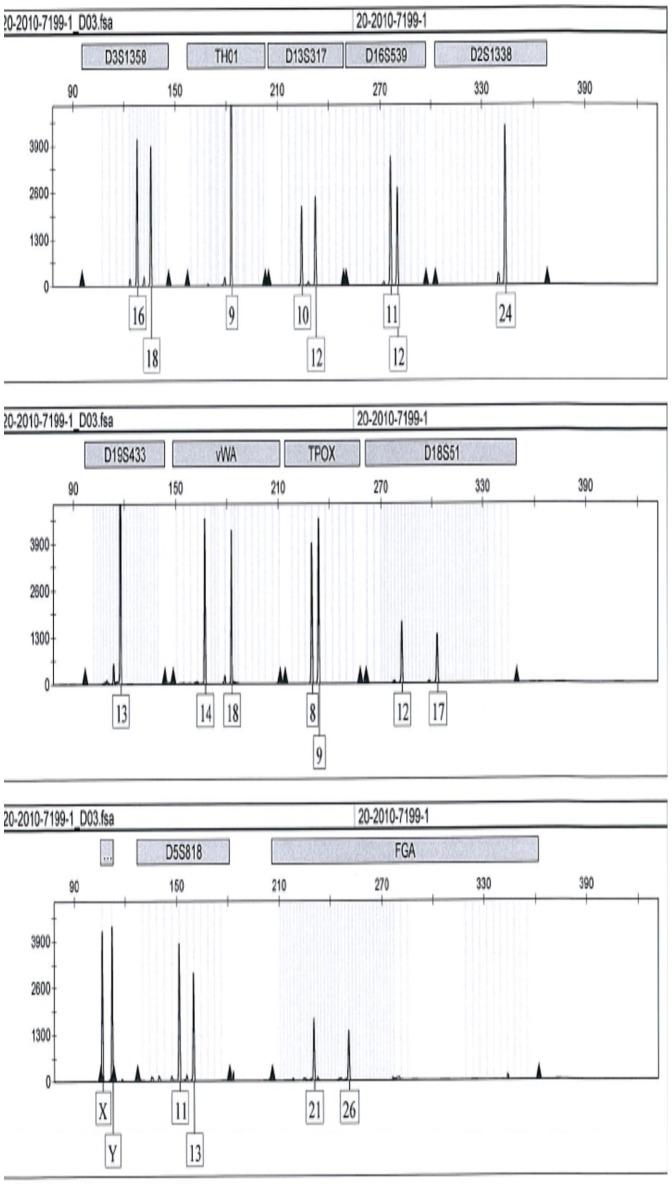
Example of a part of a DNA profile obtained from analysis of a part of a tooth root. The Identifiler™ kit is widely used and includes the profile of the amelogenin gene, here showing a result from a male subject (bottom panel).

A small part of the root of 29 teeth from 25 individuals was used for DNA analysis. A full profile was obtained for 22 of the teeth and in all cases the sex could be determined using the amelogenin gene as a marker [22], see Fig. 3. In four cases, two teeth were analyzed and the results were identical for different teeth from the same individual. Furthermore, in select cases, the results of the amelogenin gene analysis was confirmed by analysis of Y chromosome specific STR markers using Powerplex® Y System (Promega Corp., Madison, WI).

## Discussion

We report that ^14^C analysis of enamel from teeth collected from North American subjects predicts the date of birth of the individual with an average absolute error of 1.8±1.3 years. This precision is similar to that of previous studies on teeth obtained from other countries around the world [6], [23], [24], [25], [26]. Given that a large number of above ground detonations were performed in the Nevada desert, a concern regarding the reliability of this method could be raised about subjects born and raised during the cold war in nearby regions, such as California, Texas and northern Mexico. However, from Table 2 and Table S1, it can be appreciated that the precision of birth dating such individuals was not different than that of others in this study. The enamel formation times differ between different types of teeth, but also show a variation among the same type of tooth, with third molar teeth showing the highest variation [20], [27]. Despite this fact, we found that analysis of two or more teeth, including third molars, from the same individual provided similar estimates of date of birth of the individual. We also report that the ^14^C levels of two teeth from the same individual disclosed the order in which the teeth were formed, provided that their enamel formation times were sufficiently different. This can help to differentiate between teeth formed during the rising and falling part of the bomb-curve. However, using one single tooth, we also show that ^14^C analysis of both enamel and root can accomplish the differentiation. This confirms the results that Cook *et al.* (2006) reported by analysis of ^14^C in enamel and root collagen [25], [26], but shows that extraction of the collagen constitutes an unnecessary step. Recently, Kondo-Nakamura *et al*. (2010) described a different approach, using separate analysis of the occlusal and cervical part of the enamel of the same tooth [26]. However, they only describe such analysis of two teeth, and the difference in ^14^C levels in the occlusal and cervical samples were very small. It is therefore more reliable to use either two teeth with sufficiently different enamel formation times or the enamel and root from the same tooth for ^14^C analysis to determine if the tooth has been formed during the rising or falling part of the bomb-curve. In addition, cutting off the root from the crown is much easier then separating different parts of the crown.

By analyzing teeth formed before the bomb spike we could show that there is some turnover of carbon in the root after formation has been completed. Since we only measured the mineral fraction in the roots, the incorporation of ^14^C at adult ages in these roots shows that this component of the dentin and/or cementum continue to remodel, albeit at a slow pace given the relatively low ^14^C levels found. From Table 3 it can further be appreciated that the teeth formed during the rising part of the curve (except Case 36) have higher levels of ^14^C in the root than in the enamel, implying that if a continuous turnover were significant, the incorporation of the more recent atmospheric levels would have produced lower ^14^C levels in the roots. In Case 36, the actual enamel formation time was calculated to be 1961.3, i.e. shortly before the atmospheric ^14^C maximum. Given the long time period during which the roots are developed (completed about 6–7 years after the enamel [27]), most of the incorporation of ^14^C would have occurred during the falling part of the curve, explaining a lower ^14^C level in the root than in the enamel.

The principle of estimating a persońs date of birth from ^14^C analysis of teeth has hitherto used reference data on tooth formation, in turn based on repeated radiographs of children [20]. However, we have now compiled sufficient ^14^C results to calculate an average age of carbon incorporation for most types of teeth to bypass this step, so therefore we provide a reference tabulation of average ^14^C incorporation times in Table S2 to be used for future calculations. These estimates show a generally shorter time interval than the radiographic results, which may be due either to our choice of Nollás stage 4.5 as an average of the development, or explained by incorporation into enamel of some of the carbon earlier than the average radiographic picture of tooth. The radiocarbon that we measure with AMS is that incorporated in the carbonate component of the hydroxyapatite, and how this relates to the degree of radio-opacity is not known. Nonetheless, the use of crude ^14^C levels in the enamel of teeth from subjects with a known DOB simplifies the calculation and obviously provides a more precise better precision of the DOB estimate of unknown individuals (Table S2).

Studies on hair samples from subjects in different US states [18] and in different countries [15] have shown that stable isotopes generate geographical signatures. Similarly, we have recently reported that such differences can also be seen in tooth enamel [23]. In the present study we also show that ^13^C levels in tooth enamel obviously vary within a limited geographical region such as the United States and Canada. It seems likely that analysis of several stable isotopes in teeth and hair can help to determine both the earlier origin and more recent residence of an unknown dead body and thus facilitate identification work. Interestingly, the ^13^C levels in teeth from subjects raised in Mexico are the highest we have recorded; even higher than in subjects from Chile and Uruguay. The most likely explanation for this is a high dietary intake of foods based on either corn or sugar cane, or both, since they represent C4 plants that have double fixation steps in the photosynthesis system and fail to discriminate between ^13^C and ^12^C like C3 plants such as wheat, potato, rice and sugar beet [17], [28]. The ^13^C levels in tooth roots (Tables 4 and 5) were somewhat lower than in enamel from the same teeth and showed a higher variation within restricted regions, making it less reliable than enamel levels to determine geographic origin of the person. This might be explained by a more traditional diet with locally typical basic nutritional food sources during childhood than the diet during adolescence.

The ^18^O levels in tooth roots were lowest in the northwest of the United States, which is in accordance with the map of levels in tap water previously reported [19], but showed somewhat variable levels in other parts of the United States (Fig. 2). ^18^O levels in body tissues may also be dependent on food intake with a variable level of this isotope, given that a substantial part of the diet is composed of water. Such contribution to the ^18^O levels in tooth enamel may explain the smaller variation in teeth as compared to the variation in tap water, since the different components of diet may come from places at a distance from the residence - but perhaps not sufficiently remote to make a difference regarding ^13^C.

The DNA analyses indicated the correct sex in all samples analyzed. The sex determination is important to separate possible matches from missing persons lists, but also for determining the year of birth of the person, since this calculation is based on tooth formation estimates and the tooth formation times are somewhat different between sexes for several types of teeth. DNA analyses are very swift and can be performed at short notice by many laboratories worldwide. The consistent results using two different methods for sex determination in select cases support the notion that these analyses are robust. In addition, since both the Identifiler™ kit and the Powerplex® Y System provide a specific profile of the individual, such an analysis will often be needed anyway if antemortem radiographs cannot be retrieved and it seems practical and more economical to perform this analysis sooner than later. The Doe network database includes a large number of unidentified persons and different identification methods may be used to compare the characteristics of the deceased with antemortem material from the deceased or with samples from relatives. The review of this database (Table 1) revealed that the estimates of age of the subject show a great variation and that the sex was sometimes unknown. The analysis of bomb-pulse derived ^14^C provides an accurate estimate of the year of birth of the person, but only the age at death when the year of death is known. We have previously shown that addition of established methods to determine the age at death, such as aspartic acid racemization, to the year of birth as estimated by ^14^C measurement of teeth can be used to estimate the year of death of the person [24].

In conclusion, from one single tooth, the year of birth can be estimated, a clue to geographical origin can be obtained, sex can be determined and analysis of ^14^C in both enamel and root can differentiate between tooth formation during rising and falling part of the bomb-curve. We have also previously reported that a small portion of a tooth can be used for aspartic acid racemization to estimate the age at death of a person, which will allow for a calculation of the year of death, an important factor when investigating a skeletonized body.

In conclusion, a better characterization of unknown dead victims using these analyses can improve the identification work, whether it concerns a suspect homicide case or victims of a mass disaster.

## Materials and Methods

### Collection of Teeth

In total, dentists in Mexico, the United States and Canada collected 66 teeth. The teeth were either extracted for orthodontic purposes or removed due to periodontal problems. Patientś permission to use the teeth for research instead of discarding them was obtained in all cases and the study was approved by the Regional Ethics Committee at the Karolinska Intitute (No 2010/314-31/3). The tooth number, the year and month of birth, the sex, the area where the person was raised, and the date of extraction were noted and each person and tooth were given a code. All teeth were shipped to Karolinska Institute for preparation. A control determination of the tooth number was performed blindly with the assistance of three independent forensic odontologists.

### Tooth Preparation

Teeth were divided by cutting away the crown of the tooth from the root at the level of the cervical line. To isolate the enamel, the excised crown was immersed in 10 N NaOH and placed in a water-bath sonicator overnight (Branson 150) at a maximum temperature of 70°C. Approximately every 24 hrs the NaOH was replaced and the softened non-enamel structures were removed by mechanical treatment using a dental drill and blunt dissection. Next, the enamel was washed in DDH_2_O, re-submersed in 10 N NaOH and returned to the sonicator. This procedure was repeated for 3–5 days until all dentin and soft structures were completely removed. The enamel was then rinsed several times in DDH_2_O and dried at 65°C overnight. The enamel was weighed and kept sealed in a glass tube until pre-preparation for accelerator mass spectrometric (AMS) analysis. Roots were vertically divided to produce a somewhat larger piece for isotope analysis and a smaller piece for DNA analysis. The roots were then rinsed in DDH_2_O and no efforts were made to clean the root canal or to remove cementum.

### Pre-treatment for AMS and Stable Isotope Mass Spectrometry

Aliquots of the enamel samples were placed in culture tubes for pre-treatment to remove the surface carbon that may have contaminated the enamel between formation and analysis. Aliquots of 80–150 mg were used to get full sized samples for ^14^C analysis. Enamel samples were immersed in 1.0 N HCl at room temperature for 1.5 hrs, rinsed 3 times with DDH_2_O and placed on a heating block at 95°C to dry overnight. The acid pre-treatment was designed to etch the outer surface of the enamel that was exposed to the harsh base environment earlier without dissolving too much of the enamel. Each dried enamel sample was broken into 5–10 pieces and placed in an individual single-use reactor. Enamel splits in individual reaction chambers were evacuated, heated and acidified with concentrated orthophosphoric acid at 90°C to hydrolyze all mineralized carbon to CO_2_. Roots were treated with 2% NaOCl for 24 h, then rinsed 10 times with DI water, followed by treatment with 0.1 N HCl for 30 min, again rinsed 10 times with DI water and finally dried on a heating block overnight. 120 mg of the roots were then pulverized, and 1–2 mg of the powder was used for stable isotope analysis of ^13^C and ^18^O. Stable oxygen and carbon isotope ratios were determined by reaction of the powdered aliquot with supersaturated orthophosphoric acid at 90°C in an Isocarb common acid bath autocarbonate device attached to a Fisons Optima isotope ratio mass spectrometer. This method liberates carbonate so only the mineral fraction of the roots was used for stable isotope analysis.

The remainder of the powdered roots was used for AMS analysis of ^14^C and underwent similar processing as enamel after being placed in individual reactors. The evolved CO_2_ from roots and enamel was purified, trapped, and reduced to graphite in the presence of iron catalyst in individual reactors [29], [30]. The CO_2_ from large enamel samples was split prior to graphitization and δ^13^C measured by stable isotope ratio mass spectrometry. Background values were controlled by consistently following procedures, frequently baking sample tubes, periodically cleaning rigs, and maintaining a clean lab [31].

### Accelerator Mass Spectrometry Analysis

Graphite targets were measured using the 10-MV HVEE FN-class tandem electrostatic AMS system at the Center for Accelerator Mass Spectrometry at Lawrence Livermore National Laboratory (LLNL). The operation is similar to that used when performing high-precision measurements of 18,000 year old turbidities used as secondary standards [32]. The system employs a LLNL designed high-output negative ion solid graphite Cs-sputter source [33] which emits 250–350 µg of ^12^C- from a full-sized sample, corresponding to approx. 900 ^14^C counts per second from a contemporary sample. The FN AMS system routinely achieves 15% total system efficiency for C analyzing ^14^C^4+^ in the detector [34]. Details on the design of the LLNL AMS system and its operation can be found in the literature [32], [33], [34], [35]. Enamel samples are usually full sized and contemporary, so analysis times are relatively rapid, generally less than 5 minutes. The enamel samples are measured for 30,000 ^14^C counts per cycle for 5–7 cycle repetitions and achieve standard deviations of 0.3–0.8%.

Corrections for background contamination introduced during AMS sample preparation are made by establishing the contributions from contemporary and fossil carbon, following the procedures of Brown and Southon [36]. All data are normalized using six identically prepared NIST SRM 4990B (Oxalic Acid I) primary standards. NIST SRM 4990C (Oxalic Acid II), IAEA-C6 [37], and TIRI [38] wood are used as secondary standards and quality controls to monitor spectrometer performance. The ratio of NIST SRM 4990C to NIST SRM 4990B (Oxalic Acid II/Oxalic Acid I) measured between February 2005 and March 2012 on 31 different sample wheels containing enamel samples had an average value of 1.291±0.003 (1 SD), in agreement with the certified value of 1.293±0.001. ^14^C-free calcite serves as background material for processing the enamel samples. The enamel samples are organized in groups of 10–14 unknowns bracketed by primary standards with one primary standard in the middle of the group. The secondary standards, primary standards and group of unknowns are measured consecutively as a cycle. Upon completion of a cycle the set of primary standards, secondary standards and unknown samples are measured again until desired precision is achieved. A typical group of 14 enamel samples is measured completely in 2–3 h. The measurement error is determined for each sample and generally ranges between ±0.2–0.8% (1 SD). All ^14^C data are reported using the F^14^C fraction modern nomenclature developed for post-bomb data [39]. F^14^C is a concentration unit (^14^C/C) denoting enrichment or depletion of ^14^C relative to oxalic acid standard normalized for isotope fractionation. Data are also reported as decay corrected Δ^14^C following the nomenclature of Stuiver and Pollach [40]. Δ^14^C was calculated using the equation:

Δ^14^C = 1000 * ((F^14^C * exp[λ*(1950 - y)] –1)) where λ = 1/8267 yr^−1^ and y = year of measurement after 1950 A.D.

### From ^14^C to Year of Birth

The average age at which enamel formation is completed for each specific tooth has been determined previously and is dependent on the specific tooth and sex of the person [20], [27]. In cases where the sex is unknown, the average time for enamel completion for males and females can be calculated. However in this study the sex was known for all individuals and confirmed by DNA analysis. To estimate an individual’s date of birth the ^14^C concentration measured in the tooth enamel was compared with the Calibomb reference data (http://calib.qub.ac.uk/CALIBomb) or Levin and Kromer [10] to find out the year of enamel formation of the particular tooth. By subtracting the average formation time for each type of tooth [20], the individuaĺs date of birth can be calculated.

If it is not obvious whether an individual is born before or after the peak of atmospheric ^14^C levels resulting from the bomb tests, then two teeth with different enamel formation times can be analyzed – this will distinguish whether the ^14^C measurement relates to the rising or falling part of the curve. In 14 cases, two or more teeth from the same individual were subjected to AMS analysis. In addition, roots from the same teeth were also analyzed. The root of a tooth will have been formed later than the enamel and thus analysis of one single tooth can also provide such information [25], [27]. This principle is illustrated in Fig. 1.

### DNA Analysis

DNA was extracted from small fragments of the 30 roots of teeth according to a previously described method [41] for bone DNA extraction. In short: the roots were placed in 96% ethanol for a few minutes and rinsed with 0.5% Na-hypochlorite and dried overnight at 56°C in open tubes. The roots were then ground to a fine powder in liquid N_2_ in a Freezer/Mill 6850-115 (SPEX Certiprep, New Jersey). The grinding cycles were the same as for bone treatment, but grinding was shortened to only half a minute. DNA was extracted from the total amount of tooth powder using one phenol-chlorophorm and one chlorophorm extraction [42], concentrated on Centricon 30 columns (Millipore, Billerica, MA), purified using the Qiaquick Purification Kit (Qiagen, Hilden, Germany) and eluted in 45 µL buffer.

The DNA concentration was measured with a NanoDrop® 2000 (Thermo Fischer Scientific, Wilmington, USA). Duplicate aliquots of approximately 1 ng were used for the PCR amplifications according to the Identifiler™ protocol (Applied Biosystems, Foster City, CA) in a final volume of 10 µL. To verify the results in weak samples these were also amplified with a two-phase PCR protocol of 10 and 20 cycles as previously described [41]. The DNA profiles, including amelogenin, the marker for the determination of gender [43], were analyzed by capillary electrophoreses in an ABI3100 Genetic Analyzer (Applied Biosystems) and evaluated using GeneMapper ID 3.2 (Applied Biosystems). In select cases, the results of the amelogenin gene analysis was confirmed by analysis of Y chromosome specific STR markers using Powerplex® Y System (Promega Corp., Madison, WI), carried out according to the manufactureŕs manual. The DNA extraction and amplification was performed using the same PCR procedure as outlined for the Identifiler™ protocol.

### Statistical Analysis

All results are given as means ± SD. Differences between two groups were examined for statistical significance with two-tailed Student’s t test or with ANOVA, when appropriate. Significance was accepted at a *p* value of <.05.

### Ethical Considerations

The study was approved by the Regional Ethics Committee at the Karolinska Intitute (No 2010/314-31/3). The ethics committee approved this procedure provided that the patient or the caretaker gave verbal informed consent. Since dental clinics have a heavy workload, we declared that written consent would be difficult to obtain, and the ethics committee agreed. There was no documentation in the dental records that the extracted teeth were used for analysis instead of being discarded. Only the year and month of birth, and the gender were noted on the bags in which the teeth were placed.

## Supporting Information

Table S1 **Background data and all δ ^14^C and δ^ 13^C results for enamel.**
(DOC)

Table S2 **Compilation of DOB estimation using average ^14^C incorporation time interval only and using both ^14^C and enamel laydown data.**
(DOC)

Supporting Information S1 **Supporting file containing Tables S3–S5.** Table S3. Mean difference between ^14^C-measured age and Nolla's calibration. Positive values indicate that the measured age at tooth formation is larger than Nolla's estimate. Most values are negative, indicating that the incorporation of radiocarbon on average is taking place earlier than the enamel radio-opacity according to Nolla's stage 4.5 estimate. Table S4. Standard deviation in age at tooth formation for each tooth type and jaw. Table S5. Confidence intervals for the date of birth given the enamel formation year. Note that this does not use Nolla's estimates, only the ^14^C calibrated values.
(DOC)
