# Supplementary material for: Analysis of Radiocarbon, Stable Isotopes and DNA in Teeth to Facilitate Identification of Unknown Decedents
Source: PLoS One. 2013 Jul 29;8(7):e69597. doi: 10.1371/journal.pone.0069597 (PMC3726681; doi:10.1371/journal.pone.0069597)
Supplement: Table S1 — Background data and all δ 14C and δ 13C results for enamel. (DOC) [file pone.0069597.s001.doc]

**Table S1.** Background data and all δ ^14^C and δ ^13^C results for enamel.

| **Case No.** | **Sex** | **Tooth No.** | **Enamel formation time (yrs)^1^** | **Raised in / collected in** | δ **^13^C^2^** | δ **^14^C** | **±** | **Fraction modern** | **±** | **Actual tooth DOB** | **Estimated tooth DOB** | **Error in yrs (2σ)** | **Actual DOB** | **Estimated DOB** | **Error** | **Absolute error** |
| --- | --- | --- | --- | --- | --- | --- | --- | --- | --- | --- | --- | --- | --- | --- | --- | --- |
| 1 | M | 41 | 2.5 | BC | *-10.00* | 529.9 | 6.7 | 1.5410 | 0.0067 | 1965.3 | 1963.1 | 1.0 | 1962.8 | 1960.6 | -2.2 | 2.2 |
| 2 | F | 42 | 2.8 | BC | *-11.00* | -8.6 | 3.8 | 0.9986 | 0.0038 | 1918.4 | Pre-bomb | Pre-bomb | 1915.6 | Pre-bomb | Pre-bomb | Pre-bomb |
| 3 | M | 15 | 6.6 | BC | -11.78 | 525.0 | 4.5 | 1.5361 | 0.0045 | 1974.0 | 1970.1 | 0.1 | 1967.4 | 1963.5 | -3.9 | 3.9 |
| 4 | M | 44 | 5.1 | BC | -11.48 | 104.9 | 4.2 | 1.1130 | 0.0042 | 2001.7 | 1996,3 | 2.1 | 1996.6 | 1991.2 | -5.4 | 5.4 |
| 5 | F | 36 | 2.3 | BC | *-10.99* | 354.3 | 5.1 | 1.3645 | 0.0051 | 1972.6 | 1967.4 | 1.0 | 1970.4 | 1974.2 | 3.8 | 3.8 |
| 6 | M | 22 | 4.0 | BC | -10.00 | 657.4 | 7.2 | 1.6698 | 0.0072 | 1966.2 | 1966.8 | 0.4 | 1962.2 | 1962.9 | 0.7 | 0.7 |
| 7 | M | 38 | 13.0 | WA | *-10.00* | 127.3 | 4.0 | 1.1357 | 0.0040 | 1995.3 | 1992.5 | 1.7 | 1982.3 | 1979.5 | -2.8 | 2.8 |
| 7 | M | 48 | 13.0 | WA | *-10.00* | 124.0 | 4.2 | 1.1323 | 0.0042 | 1995.3 | 1993.1 | 1.9 | 1982.3 | 1980.1 | -2.2 | 2.2 |
| 8 | F | 27 | 5.8 | WA | *-10.00* | 633.8 | 5.9 | 1.6459 | 0.0059 | 1968.6 | 1967.2 | 0.3 | 1962.8 | 1961.4 | -1.4 | 1.4 |
| 8 | F | 45 | 5.7 | WA | *-10.00* | 623.6 | 5.7 | 1.6356 | 0.0057 | 1968.5 | 1967.4 | 0.3 | 1962.8 | 1961.7 | -1.1 | 1.1 |
| 9 | M | 17 | 6.5 | WA | *-10.00* | 173.3 | 4.2 | 1.1820 | 0.0042 | 1989.8 | 1987.5 | 1.4 | 1983.3 | 1981.0 | -2.3 | 2.3 |
| 10 | M | 47 | 6.5 | WA | *-10.00* | 332.3 | 4.7 | 1.3421 | 0.0047 | 1979.9 | 1977.5 | 1.0 | 1973.4 | 1971.0 | -2.4 | 2.4 |
| 11 | F | 24 | 4.9 | WA | *-10.00* | 348.5 | 5.0 | 1.3585 | 0.0050 | 1964.9 | 1962.4 | 0.2 | 1960.0 | 1957.5 | -2.5 | 2.5 |
| 12 | M | 16 | 3.3 | WA | *-10.00* | 478.8 | 5.3 | 1.4898 | 0.0053 | 1973.9 | 1971.8 | 0.5 | 1970.6 | 1968.5 | -2.1 | 2.1 |
| 12 | M | 37 | 6.5 | WA | *-10.00* | 341.9 | 4.8 | 1.3518 | 0.0048 | 1977.1 | 1976.8 | 1.0 | 1970.6 | 1970.3 | -0.3 | 0.3 |
| 13 | M | 15 | 6.6 | WA | *-10.00* | 108.8 | 4.0 | 1.1170 | 0.0040 | 1998.4 | 1995.6 | 1.9 | 1991.8 | 1989.0 | -2.8 | 2.8 |
| 14 | M | 17 | 6.5 | MT | *-10.00* | 156.1 | 4.1 | 1.1646 | 0.0041 | 1991.0 | 1989.4 | 0.5 | 1984.5 | 1982.9 | -1.6 | 1.6 |
| 14 | M | 18 | 12.6 | MT | *-10.00* | 115.9 | 6.0 | 1.1242 | 0.0060 | 1997.1 | 1994.3 | 2.4 | 1984.5 | 1981.7 | -2.8 | 2.8 |
| 14 | M | 28 | 12.6 | MT | *-10.00* | 103.6 | 3.9 | 1.1118 | 0.0039 | 1997.1 | 1996.5 | 2.1 | 1984.5 | 1983.9 | -0.6 | 0.6 |
| 15 | F | 25 | 5.6 | MT | *-10.00* | 357.5 | 4.8 | 1.3675 | 0.0048 | 1975.4 | 1975.8 | 0.8 | 1969.8 | 1970.2 | 0.4 | 0.4 |
| 15 | F | 26 | 3.0 | MT | *-10.00* | 464.1 | 5.2 | 1.4750 | 0.0052 | 1972.8 | 1972.2 | 0.4 | 1969.8 | 1969.2 | -0.6 | 0.6 |
| 17 | M | 45 | 6.5 | MA | *-7.89* | 267.7 | 3.6 | 1.2773 | 0.0036 | 1964.2 | 1962.0 | 0.2 | 1957.7 | 1956.0 | -1.7 | 1.7 |
| 18 | M | 14 | 5.6 | CT | -10.82 | 84.3 | 3.1 | 1.0922 | 0.0031 | 2002.8 | 2000.0 | 1.8 | 1997.2 | 1994.4 | -2.8 | 2.8 |
| 18 | M | 44 | 5.1 | CT | *-10.00* | 86.1 | 3.9 | 1.0940 | 0.0039 | 2002.3 | 2000.0 | 1.9 | 1997.2 | 1994.9 | -2.3 | 2.3 |
| 19 | M | 14 | 5.6 | CT | -10.84 | 89.2 | 3.8 | 1.0971 | 0.0038 | 2001.9 | 1999.2 | 1.9 | 1996.3 | 1993.6 | -2.7 | 2.7 |
| 19 | M | 34 | 5.1 | CT | -10.56 | 79.4 | 3.8 | 1.0872 | 0.0038 | 2001.4 | 2001.0 | 1.1 | 1996.3 | 1995.9 | -0.4 | 0.4 |
| 16 | F | 26 | 3.0 | WA^3^ | *-10.00* | 167.9 | 4.6 | 1.1765 | 0.0046 | 1990.6 | 1988.0 | 1.5 | 1987.6 | 1985.0 | -2.6 | 2.6 |
| 20 | F | 31 | 2.5 | CA | *-10.00* | 6.1 | 3.6 | 1.0134 | 0.0036 | 1947.4 | Pre-bomb | Pre-bomb | 1944.9 | Pre-bomb | Pre-bomb | Pre-bomb |
| 20 | F | 33 | 4.1 | CA | -10.39 | -15.2 | 2.9 | 0.9920 | 0.0029 | 1949.0 | Pre-bomb | Pre-bomb | 1944.9 | Pre-bomb | Pre-bomb | Pre-bomb |
| 21 | M | 15 | 6.6 | CA | -10.51 | 42.0 | 4.4 | 1.0496 | 0.0044 | 1959.7 | 1957.3 | 0.5 | 1953.1 | 1950.7 | -2.4 | 2.4 |
| 22 | M | 28 | 12.6 | CA | *-10.00* | 137.8 | 4.0 | 1.1462 | 0.0040 | 1992.8 | 1991.6 | 1.4 | 1980.2 | 1979.0 | -1.2 | 1.2 |
| 23 | M | 42 | 3.0 | GA | *-8.98* | 218.9 | 3.8 | 1.2281 | 0.0038 | 1984.1 | 1983.3 | 0.8 | 1981.1 | 1980.3 | -0.7 | 0.7 |
| 24 | F | 23 | 4.7 | AL | *-10.00* | 9.6 | 3.6 | 1.0171 | 0.0036 | 1958.1 | 1956.1 | 0.5 | 1953.4 | 1951.4 | -2.0 | 2.0 |
| 25 | F | 14 | 4.9 | TX | -9.54 | 131.3 | 4.0 | 1.1396 | 0.0040 | 1996.3 | 1992.1 | 1.5 | 1991.4 | 1987.2 | -4.2 | 4.2 |
| 25 | F | 34 | 4.4 | TX | -9.18 | 114.2 | 3.2 | 1.1224 | 0.0032 | 1995.8 | 1994.6 | 1.5 | 1991.4 | 1990.2 | -1.2 | 1.2 |
| 26 | M | 14 | 5.6 | TX | -8.83 | -12.4 | 2.8 | 0.9948 | 0.0028 | 1945.9 | Pre-bomb | Pre-bomb | 1940.3 | Pre-bomb | Pre-bomb | Pre-bomb |
| 26 | M | 31 | 2.5 | TX | *-9.00* | -17.2 | 3.2 | 0.9900 | 0.0032 | 1942.8 | Pre-bomb | Pre-bomb | 1940.3 | Pre-bomb | Pre-bomb | Pre-bomb |
| 27 | M | 23 | 4.7 | TX | -8.41 | 164.1 | 3.6 | 1.1726 | 0.0036 | 1960.8 | 1958,8 | 0.5 | 1956.1 | 1954.1 | -2.0 | 2.0 |
| 28 | M | 36 | 2.4 | TX | -10.19 | 93.9 | 3.5 | 1.1019 | 0.0035 | 1994.4 | 1998.2 | 2.2 | 1992.0 | 1995.8 | 3.8 | 3.8 |
| 29 | M | 18 | 12.6 | TX | -8.85 | 370.0 | 3.8 | 1.3799 | 0.0038 | 1976.5 | 1975.4 | 0.7 | 1965.3 | 1962.8 | -2.5 | 2.5 |
| 29 | M | 46 | 2.4 | TX | -8.96 | 397.3 | 4.1 | 1.4075 | 0.0041 | 1967.6 | 1962.7 | 0.2 | 1965.3 | 1960.3 | -5.0 | 5.0 |
| 30 | M | 12 | 4.0 | TX | -9.04 | 299.2 | 3.8 | 1.3087 | 0.0038 | 1964.1 | 1962.1 | 0.1 | 1960.1 | 1958.1 | -2.0 | 2.0 |
| 30 | M | 13 | 4.7 | TX | *-9.00* | 340.4 | 4.1 | 1.3502 | 0.0041 | 1964.8 | 1962.4 | 0.2 | 1960.1 | 1957.7 | -2.4 | 2.4 |
| 30 | M | 14 | 5.6 | TX | *-9.00* | 463.8 | 6.2 | 1.4744 | 0.0062 | 1965.7 | 1962.9 | 0.4 | 1960.1 | 1957.3 | -2.8 | 2.8 |
| 30 | M | 22 | 4.0 | TX | *-9.00* | 605.5 | 6.6 | 1.6172 | 0.0066 | 1964.1 | 1963.3 | 0.3 | 1960.1 | 1959.3 | -0.8 | 0.8 |
| 30 | M | 23 | 4.7 | TX | *-9.00* | 727.3 | 26.7 | 1.7399 | 0.0267 | 1964.8 | 1965.8 | 1.0 | 1960.1 | 1961.1 | 1.0 | 1.0 |
| 30 | M | 26 | 3.3 | TX | -9.20 | 216.5 | 4.6 | 1.2254 | 0.0046 | 1963.4 | 1961.1 | 0.5 | 1960.1 | 1957.8 | -2.3 | 2.3 |
| 30 | M | 31 | 2.5 | TX | *-9.00* | 269.9 | 7.3 | 1.2791 | 0.0073 | 1962.6 | 1962,0 | 0.4 | 1960.1 | 1959.5 | -0.6 | 0.6 |
| 30 | M | 32 | 3.0 | TX | *-9.00* | 645.2 | 169.3 | 1.6571 | 0.1693 | 1963.1 | 1963.3 | 0.5 | 1960.1 | 1960.3 | 0.2 | 0.2 |
| 30 | M | 37 | 6.5 | TX | *-9.00* | 661.4 | 5.6 | 1.6735 | 0.0056 | 1966.6 | 1966.8 | 0.4 | 1960.1 | 1960.3 | 0.2 | 0.2 |
| 30 | M | 41 | 2.5 | TX | *-9.00* | 402.8 | 5.9 | 1.4131 | 0.0059 | 1962.6 | 1962.6 | 0.3 | 1960.1 | 1960.1 | 0.0 | 0.0 |
| 30 | M | 42 | 3.0 | TX | *-9.00* | 494.3 | 150.4 | 1.5051 | 0.1504 | 1963.1 | 1962.8 | 0.6 | 1960.1 | 1959.8 | -0.3 | 0.3 |
| 30 | M | 43 | 4.3 | TX | *-9.00* | 437.1 | 6.2 | 1.4475 | 0.0062 | 1964.4 | 1962.8 | 0.2 | 1960.1 | 1958.5 | -1.6 | 1.6 |
| 30 | M | 44 | 5.1 | TX | *-9.00* | 654.6 | 6.7 | 1.6666 | 0.0067 | 1965.2 | 1966.4 | 0.1 | 1960.1 | 1961.3 | 1.2 | 1.2 |
| 30 | M | 47 | 6.5 | TX | *-9.00* | 682.3 | 6.0 | 1.6945 | 0.0060 | 1966.6 | 1966.5 | 0.4 | 1960.1 | 1960.0 | -0.1 | 0.1 |
| 31 | M | 11 | 3.2 | TX | *-10.00* | 340.3 | 4.8 | 1.3500 | 0.0048 | 1979.6 | 1977.0 | 1.0 | 1976.4 | 1973.8 | -2.6 | 2.6 |
| 32 | F | 46 | 2.4 | Mexico | *-10.00* | -22.3 | 3.5 | 0.9849 | 0.0035 | 1946.9 | Pre-bomb | Pre-bomb | 1944.5 | Pre-bomb | Pre-bomb | Pre-bomb |
| 33 | M | 23 | 4.7 | Mexico | *-10.00* | 20.4 | 4.0 | 1.0280 | 0.0040 | 1956.8 | 1956.0 | 0.5 | 1952.1 | 1951.3 | -0.8 | 0.8 |
| 34 | M | 26 | 3.3 | Mexico | *-10.00* | 207.4 | 4.5 | 1.2163 | 0.0045 | 1962.8 | 1960.0 | 1.4 | 1959.5 | 1956.7 | -2.8 | 2.8 |
| 35 | F | 46 | 2.3 | Mexico | *-10.00* | -12.8 | 3.5 | 0.9945 | 0.0035 | 1945.7 | Pre-bomb | Pre-bomb | 1943.4 | Pre-bomb | Pre-bomb | Pre-bomb |
| 36 | M | 37 | 6.5 | Mexico | *-10.00* | 645.1 | 5.9 | 1.6573 | 0.0059 | 1961.3 | 1962.7 | 0.5 | 1954.8 | 1956.2 | 1.4 | 1.4 |
| 36 | M | 36 | 2.4 | Mexico | *-10.00* | 65.9 | 3.8 | 1.0738 | 0.0038 | 1957.2 | 1957.4 | 0.5 | 1954.8 | 1955.0 | 0.2 | 0.2 |
| 37 | F | 35 | 5.7 | Mexico | *-10.00* | -15.3 | 3.3 | 0.9920 | 0.0033 | 1926.7 | Pre-bomb | Pre-bomb | 1921.0 | Pre-bomb | Pre-bomb | Pre-bomb |
| 38 | F | 36 | 2.3 | Mexico | *-10.00* | -12.4 | 3.4 | 0.9949 | 0.0034 | 1933.5 | Pre-bomb | Pre-bomb | 1931.2 | Pre-bomb | Pre-bomb | Pre-bomb |
| 39 | F | 17 | 5.8 | Mexico | *-10.00* | 519.3 | 5.4 | 1.5306 | 0.0054 | 1969.6 | 1970.1 | 0.5 | 1963.8 | 1964.3 | 0.5 | 0.5 |
| 39 | F | 45 | 5.7 | Mexico | *-10.00* | 521.9 | 5.8 | 1.5331 | 0.0058 | 1969.5 | 1970.2 | 0.8 | 1963.8 | 1964.5 | 0.7 | 0.7 |
|  |  |  |  |  |  |  |  |  |  |  |  |  |  |  |  |  |
|  |  |  |  |  |  |  |  |  |  |  |  |  |  |  |  |  |
|  |  |  |  |  |  |  |  |  |  |  |  |  |  |  | **Average** | **1.9** |
|  |  |  |  |  |  |  |  |  |  |  |  |  |  |  | SD | 1.3 |

^1^Enamel formation time according to Nolla [20].

^2^Values in italics are estimates for each region.

^3^This person was born in England, UK, but moved to WA, unclear at what age.
