# Supplementary material for: Analysis of Radiocarbon, Stable Isotopes and DNA in Teeth to Facilitate Identification of Unknown Decedents
Source: PLoS One. 2013 Jul 29;8(7):e69597. doi: 10.1371/journal.pone.0069597 (PMC3726681; doi:10.1371/journal.pone.0069597)
Supplement: Table S2 — Compilation of DOB estimation using average 14C incorporation time interval only and using both 14C and enamel laydown data. (DOC) [file pone.0069597.s002.doc]

**Table S2.** Compilation of DOB estimation using average ^14^C incorporation time interval only and using both ^14^C and enamel laydown data.

| **Sex** | **Country^1^** | **Tooth No.** | **Actual DOB of person** | **Enamel formation time (yrs)^2^** | **F^14^C** | **+/-** | **Estimated tooth DOB using only ^14^C data^3^** | **Interval DOB - ^14^C incorpo-ration** | **Estimated DOB using enamel formation time** | **Estimated DOB using only ^14^C data** | **Error using enamel formation time** | **Absolute error using enamel formation time** | **Error using only ^14^C data** | **Absolute error using only ^14^C data** |
| --- | --- | --- | --- | --- | --- | --- | --- | --- | --- | --- | --- | --- | --- | --- |
| M | TX | 11 | 1976.4 | 3.2 | 1.3500 | 0.0048 | 1977.0 | 0.6 | 1973.8 | 1974.8 | -2.6 | 2.6 | -1.6 | 1.6 |
| F | n/a | 11 | 1957.7 | 3.2 | 1.1310 | 0.0070 | 1959.8 | 2.1 | 1956.6 | 1957.6 | -1.1 | 1.1 | -0.1 | 0.1 |
| M | Sweden | 21 | 1982.0 | 3.2 | 1.2138 | 0.0031 | 1984.4 | 2.4 | 1981.2 | 1982.2 | -0.8 | 0.8 | 0.2 | 0.2 |
| M | Lebanon | 21 | 1962.6 | 3.2 | 1.5237 | 0.0164 | 1963.0 | 0.4 | 1959.8 | 1960.8 | -2.8 | 2.8 | -1.8 | 1.8 |
| F | n/a | 21 | 1957.7 | 3.2 | 1.1420 | 0.0070 | 1959.8 | 2.1 | 1956.6 | 1957.6 | -1.1 | 1.1 | -0.1 | 0.1 |
| F | Scand? | 21 | 1970.4 | 3.2 | 1.3920 | 0.0060 | 1974.8 | 4.4 | 1971.6 | 1972.6 | 1.2 | 1.2 | 2.2 | 2.2 |
| F | n/a | 21 | 1975.0 | 3.2 | 1.3150 | 0.0060 | 1978.8 | 3.8 | 1975.6 | 1976.6 | 0.6 | 0.6 | 1.6 | 1.6 |
| F | Poland | 11 | 1957.0 | 3.2 | 1.1471 | 0.0045 | 1958.5 | 1.5 | 1955.3 | 1956.3 | -1.7 | 1.7 | -0.7 | 0.7 |
|  |  |  | **Mean** | **3.2** |  |  | **Mean** | **2.2** |  | **Mean** | **-1.0** | **1.5** | **0.0** | **1.0** |
|  |  |  |  |  |  |  | **SD** | **1.4** |  | **SD** | **1.4** | **0.8** | **1.4** | **0.9** |
|  |  |  |  |  |  |  |  |  |  |  |  |  |  |  |
| M | TX | 12 | 1960.1 | 4.0 | 1.3087 | 0.0038 | 1962.1 | 2.0 | 1958.2 | 1958.4 | -1.9 | 1.9 | -1.8 | 1.8 |
| F | n/a | 12 | 1990.5 | 3.7 | 1.1200 | 0.0040 | 1995.2 | 4.7 | 1991.3 | 1991.5 | 0.8 | 0.8 | 1.0 | 1.0 |
| F | n/a | 12 | 1975.0 | 3.7 | 1.2930 | 0.0060 | 1979.7 | 4.7 | 1975.8 | 1976.0 | 0.8 | 0.8 | 1.0 | 1.0 |
| F | n/a | 12 | 1971.8 | 3.7 | 1.4010 | 0.0080 | 1974.4 | 2.6 | 1970.5 | 1970.7 | -1.3 | 1.3 | -1.1 | 1.1 |
| M | TX | 22 | 1960.1 | 4.0 | 1.6172 | 0.0066 | 1963.3 | 3.2 | 1959.4 | 1959.6 | -0.7 | 0.7 | -0.5 | 0.5 |
| F | n/a | 22 | 1975.0 | 3.7 | 1.3100 | 0.0060 | 1979.0 | 4.0 | 1975.1 | 1975.3 | 0.1 | 0.1 | 0.3 | 0.3 |
| M | Bosnia | 22 | 1953.0 | 4.0 | 1.0471 | 0.0038 | 1957.2 | 4.2 | 1953.3 | 1953.5 | 0.3 | 0.3 | 0.5 | 0.5 |
| M | BC | 22 | 1962.2 | 4.0 | 1.6698 | 0.0072 | 1966.8 | 4.6 | 1962.9 | 1963.1 | 0.7 | 0.7 | 0.8 | 0.8 |
|  |  |  | **Mean** | **3.9** |  |  | **Mean** | **3.8** |  | **Mean** | **-0.2** | **0.8** | **0.0** | **0.9** |
|  |  |  |  |  |  |  | **SD** | **1.0** |  | **SD** | **1.0** | **0.6** | **1.0** | **0.5** |
|  |  |  |  |  |  |  |  |  |  |  |  |  |  |  |
| F | Japan | 13 | 1967.3 | 3.8 | 1.5434 | 0.0060 | 1970.1 | 2.8 | 1965.7 | 1966.8 | -1.6 | 1.6 | -0.5 | 0.5 |
| F | n/a | 13 | 1990.5 | 3.8 | 1.1200 | 0.0050 | 1995.3 | 4.8 | 1990.9 | 1992.0 | 0.4 | 0.4 | 1.5 | 1.5 |
| M | TX | 13 | 1960.1 | 4.7 | 1.3502 | 0.0041 | 1962.4 | 2.3 | 1958.0 | 1959.1 | -2.1 | 2.1 | -1.0 | 1.0 |
| F | AL | 23 | 1953.4 | 4.7 | 1.0171 | 0.0036 | 1956.1 | 2.7 | 1951.7 | 1952.8 | -1.7 | 1.7 | -0.6 | 0.6 |
| M | Mexico | 23 | 1952.1 | 4.7 | 1.0280 | 0.0040 | 1956.0 | 3.9 | 1951.6 | 1952.7 | -0.5 | 0.5 | 0.6 | 0.6 |
| M | TX | 23 | 1956.1 | 4.7 | 1.1726 | 0.0036 | 1958.8 | 2.7 | 1954.4 | 1955.5 | -1.7 | 1.7 | -0.6 | 0.6 |
| M | TX | 23 | 1960.1 | 4.7 | 1.7399 | 0.0267 | 1965.8 | 5.7 | 1961.4 | 1962.5 | 1.3 | 1.3 | 2.4 | 2.4 |
| F | Poland | 13 | 1957.0 | 3.8 | 1.1403 | 0.0041 | 1958.4 | 1.4 | 1954.0 | 1955.1 | -3.0 | 3.0 | -1.9 | 1.9 |
|  |  |  | **Mean** | **4.4** |  |  | **Mean** | **3.3** |  | **Mean** | **-1.1** | **1.5** | **0.0** | **1.1** |
|  |  |  |  |  |  |  | **SD** | **1.3** |  | **SD** | **1.4** | **0.8** | **1.4** | **0.7** |
|  |  |  |  |  |  |  |  |  |  |  |  |  |  |  |
| F | n/a | 14 | 1991.5 | 4.9 | 1.1251 | 0.0072 | 1994.2 | 2.7 | 1989.0 | 1990.6 | -2.5 | 2.5 | -0.9 | 0.9 |
| F | Sweden | 14 | 1988.8 | 4.9 | 1.1533 | 0.0032 | 1990.6 | 1.8 | 1985.4 | 1987.0 | -3.4 | 3.4 | -1.8 | 1.8 |
| F | Sweden | 14 | 1991.3 | 4.9 | 1.1088 | 0.0040 | 1997.2 | 5.9 | 1992.0 | 1993.6 | 0.7 | 0.7 | 2.3 | 2.3 |
| F | Sweden | 14 | 1989.1 | 4.9 | 1.1390 | 0.0045 | 1992.2 | 3.1 | 1987.0 | 1988.6 | -2.1 | 2.1 | -0.5 | 0.5 |
| F | Sweden | 14 | 1991.1 | 4.9 | 1.1195 | 0.0041 | 1995.2 | 4.1 | 1990.0 | 1991.6 | -1.1 | 1.1 | 0.5 | 0.5 |
| F | Sweden | 14 | 1991.5 | 4.9 | 1.1177 | 0.0041 | 1995.5 | 4.0 | 1990.3 | 1991.9 | -1.2 | 1.2 | 0.4 | 0.4 |
| F | Sweden | 14 | 1988.3 | 4.9 | 1.1413 | 0.0037 | 1992.0 | 3.7 | 1986.8 | 1988.4 | -1.5 | 1.5 | 0.1 | 0.1 |
| F | TX | 14 | 1991.4 | 4.9 | 1.1396 | 0.0040 | 1992.1 | 0.7 | 1986.9 | 1988.5 | -4.5 | 4.5 | -2.9 | 2.9 |
| M | Chile | 14 | 1967.8 | 5.6 | 1.4825 | 0.0057 | 1972.0 | 4.2 | 1966.8 | 1968.4 | -1.0 | 1.0 | 0.6 | 0.6 |
| M | Sweden | 14 | 1993.2 | 5.6 | 1.1024 | 0.0042 | 1998.1 | 4.9 | 1992.9 | 1994.5 | -0.3 | 0.3 | 1.3 | 1.3 |
| M | Sweden | 14 | 1990.9 | 5.6 | 1.1217 | 0.0043 | 1994.9 | 4.0 | 1989.7 | 1991.3 | -1.2 | 1.2 | 0.4 | 0.4 |
| M | Japan | 14 | 1964.2 | 5.6 | 1.5797 | 0.0061 | 1968.4 | 4.2 | 1963.2 | 1964.8 | -1.0 | 1.0 | 0.6 | 0.6 |
| M | CT | 14 | 1997.2 | 5.6 | 1.0922 | 0.0031 | 2000.0 | 2.8 | 1994.8 | 1996.4 | -2.4 | 2.4 | -0.8 | 0.8 |
| M | CT | 14 | 1996.3 | 5.6 | 1.0971 | 0.0038 | 1999.2 | 2.9 | 1994.0 | 1995.6 | -2.3 | 2.3 | -0.7 | 0.7 |
| M | TX | 14 | 1960.1 | 5.6 | 1.4744 | 0.0062 | 1962.9 | 2.8 | 1957.7 | 1959.3 | -2.4 | 2.4 | -0.8 | 0.8 |
| M | Sweden | 14 | 1990.8 | 5.6 | 1.1196 | 0.0043 | 1995.3 | 4.5 | 1990.1 | 1991.7 | -0.7 | 0.7 | 0.9 | 0.9 |
| F | WA | 24 | 1960.0 | 4.9 | 1.3585 | 0.0050 | 1962.4 | 2.4 | 1957.2 | 1958.8 | -2.8 | 2.8 | -1.2 | 1.2 |
| F | Sweden | 24 | 1990.5 | 4.9 | 1.1215 | 0.0054 | 1994.9 | 4.4 | 1989.7 | 1991.3 | -0.8 | 0.8 | 0.8 | 0.8 |
| F | Sweden | 24 | 1991.8 | 4.9 | 1.1133 | 0.0042 | 1996.2 | 4.4 | 1991.0 | 1992.6 | -0.8 | 0.8 | 0.8 | 0.8 |
| M | n/a | 24 | 1992.1 | 5.6 | 1.1198 | 0.0050 | 1995.3 | 3.2 | 1990.1 | 1991.7 | -2.0 | 2.0 | -0.4 | 0.4 |
| M | Scand? | 24 | 1993.1 | 5.6 | 1.1030 | 0.0040 | 1998.0 | 4.9 | 1992.8 | 1994.4 | -0.3 | 0.3 | 1.3 | 1.3 |
|  |  |  | **Mean** | **5.2** |  |  | **Mean** | **3.6** |  | **Mean** | **-1.6** | **1.7** | **0.0** | **1.0** |
|  |  |  |  |  |  |  | **SD** | **1.2** |  | **SD** | **1.2** | **1.1** | **1.2** | **0.7** |
|  |  |  |  |  |  |  |  |  |  |  |  |  |  |  |
| F | N | 15 | 1989.1 | 5.6 | 1.1301 | 0.0039 | 1993.4 | 4.3 | 1987.2 | 1988.6 | -1.9 | 1.9 | -0.5 | 0.5 |
| F | Scand? | 15 | 1988.6 | 5.6 | 1.1264 | 0.0043 | 1993.8 | 5.2 | 1987.6 | 1989.0 | -1.0 | 1.0 | 0.4 | 0.4 |
| F | Na | 15 | 1989.5 | 5.6 | 1.1120 | 0.0040 | 1996.5 | 7.0 | 1990.3 | 1991.7 | 0.8 | 0.8 | 2.2 | 2.2 |
| M | BC | 15 | 1967.4 | 6.6 | 1.5361 | 0.0045 | 1970.1 | 2.7 | 1963.9 | 1965.3 | -3.5 | 3.5 | -2.1 | 2.1 |
| M | CA | 15 | 1953.1 | 6.6 | 1.0496 | 0.0044 | 1957.3 | 4.2 | 1951.1 | 1952.5 | -2.0 | 2.0 | -0.6 | 0.6 |
| M | WA | 15 | 1991.8 | 6.6 | 1.1170 | 0.0040 | 1995.6 | 3.8 | 1989.4 | 1990.8 | -2.4 | 2.4 | -1.0 | 1.0 |
| M | Chile | 15 | 1967.8 | 6.6 | 1.4785 | 0.0236 | 1972.1 | 4.3 | 1965.9 | 1967.3 | -1.9 | 1.9 | -0.5 | 0.5 |
| M | Uruguay | 25 | 1977.4 | 6.6 | 1.2275 | 0.0044 | 1983.4 | 6.0 | 1977.2 | 1978.6 | -0.2 | 0.2 | 1.2 | 1.2 |
| F | MT/WA | 25 | 1969.8 | 5.6 | 1.3675 | 0.0048 | 1975.8 | 6.0 | 1969.6 | 1971.0 | -0.2 | 0.2 | 1.2 | 1.2 |
|  |  |  | **Mean** | **6.2** |  |  | **Mean** | **4.8** |  | **Mean** | **-1.4** | **1.5** | **0.0** | **1.1** |
|  |  |  |  |  |  |  | **SD** | **1.3** |  | **SD** | **1.3** | **1.1** | **1.3** | **0.7** |
|  |  |  |  |  |  |  |  |  |  |  |  |  |  |  |
| M | WA | 16 | 1970.6 | 3.3 | 1.4898 | 0.0053 | 1971.8 | 1.2 | 1968.6 | 1970.3 | -2.0 | 2.0 | -0.3 | 0.3 |
| M | TX | 26 | 1960.1 | 3.3 | 1.2254 | 0.0046 | 1961.1 | 1.0 | 1957.9 | 1959.6 | -2.2 | 2.2 | -0.5 | 0.5 |
| M | Chile | 26 | 1967.8 | 3.3 | 1.5173 | 0.0108 | 1970.8 | 3.0 | 1967.6 | 1969.3 | -0.2 | 0.2 | 1.5 | 1.5 |
| M | Scand? | 26 | 1956.5 | 3.3 | 1.0543 | 0.0036 | 1957.1 | 0.6 | 1953.9 | 1955.6 | -2.6 | 2.6 | -0.9 | 0.9 |
| M | Mexico | 26 | 1959.5 | 3.3 | 1.2163 | 0.0045 | 1960.0 | 0.5 | 1956.8 | 1958.5 | -2.7 | 2.7 | -1.0 | 1.0 |
| F | Uruguay | 26 | 1995.2 | 3.0 | 1.1137 | 0.0036 | 1996.1 | 0.9 | 1992.9 | 1994.6 | -2.3 | 2.3 | -0.6 | 0.6 |
| F | England/WA^4^ | 26 | 1987.6 | 3.0 | 1.1765 | 0.0046 | 1988.0 | 0.4 | 1984.8 | 1986.5 | -2.8 | 2.8 | -1.1 | 1.1 |
| F | MT/WA | 26 | 1969.8 | 3.0 | 1.4750 | 0.0052 | 1972.2 | 2.4 | 1969.0 | 1970.7 | -0.8 | 0.8 | 0.9 | 0.9 |
| F |  | 16 | 1955.1 | 3.0 | 1.0089 | 0.0037 | 1958.7 | 3.6 | 1955.5 | 1957.2 | 0.4 | 0.4 | 2.1 | 2.1 |
|  |  |  | **Mean** | **3.2** |  |  | **Mean** | **1.5** |  | **Mean** | **-1.7** | **1.8** | **0.0** | **1.0** |
|  |  |  |  |  |  |  | **SD** | **1.2** |  | **SD** | **1.2** | **1.0** | **1.2** | **0.5** |
|  |  |  |  |  |  |  |  |  |  |  |  |  |  |  |
| F | N | 17 | 1972.2 | 5.8 | 1.3359 | 0.0042 | 1977.8 | 5.6 | 1971.7 | 1972.7 | -0.5 | 0.5 | 0.5 | 0.5 |
| F | Sweden | 17 | 1993.4 | 5.8 | 1.1035 | 0.0036 | 1998.1 | 4.7 | 1992.0 | 1993.0 | -1.4 | 1.4 | -0.4 | 0.4 |
| F | Sweden | 17 | 1963.7 | 5.8 | 1.5653 | 0.0051 | 1969.4 | 5.7 | 1963.3 | 1964.3 | -0.4 | 0.4 | 0.6 | 0.6 |
| F | Scand? | 17 | 1955.8 | 5.8 | 1.2160 | 0.0038 | 1960.0 | 4.2 | 1953.9 | 1954.9 | -1.9 | 1.9 | -0.9 | 0.9 |
| F | Mexico | 17 | 1963.8 | 5.8 | 1.5306 | 0.0054 | 1970.1 | 6.3 | 1964.0 | 1965.0 | 0.2 | 0.2 | 1.2 | 1.2 |
| M | MT/WA | 17 | 1984.5 | 6.5 | 1.1646 | 0.0041 | 1989.4 | 4.9 | 1983.3 | 1984.3 | -1.2 | 1.2 | -0.2 | 0.2 |
| M | WA | 17 | 1983.3 | 6.5 | 1.1820 | 0.0042 | 1987.5 | 4.2 | 1981.4 | 1982.4 | -1.9 | 1.9 | -0.9 | 0.9 |
| M | Scand? | 17 | 1952.3 | 6.5 | 1.0277 | 0.0026 | 1956.4 | 4.1 | 1950.3 | 1951.3 | -2.0 | 2.0 | -1.0 | 1.0 |
| M | Iraq | 27 | 1975.8 | 6.5 | 1.2706 | 0.0042 | 1980.8 | 5.0 | 1974.7 | 1975.7 | -1.1 | 1.1 | -0.1 | 0.1 |
| M | Scand? | 27 | 1989.6 | 6.5 | 1.1167 | 0.0038 | 1997.0 | 7.4 | 1990.9 | 1991.9 | 1.3 | 1.3 | 2.3 | 2.3 |
| F | WA | 27 | 1962.8 | 5.8 | 1.6459 | 0.0059 | 1967.2 | 4.4 | 1961.1 | 1962.1 | -1.7 | 1.7 | -0.7 | 0.7 |
|  |  |  | **Mean** | **6.1** |  |  | **Mean** | **5.1** |  | **Mean** | **-1.0** | **1.2** | **0.0** | **0.8** |
|  |  |  |  |  |  |  | **SD** | **1.0** |  | **SD** | **1.0** | **0.6** | **1.0** | **0.6** |
|  |  |  |  |  |  |  |  |  |  |  |  |  |  |  |
|  |  |  |  |  |  |  |  |  |  |  |  |  |  |  |
| F | Scand? | 18 | 1965.3 | 11.2 | 1.3065 | 0.0038 | 1979.2 | 13.9 | 1967.1 | 1967.7 | 1.8 | 1.8 | 2.4 | 2.4 |
| M | Scand? | 18 | 1962.6 | 12.6 | 1.4280 | 0.0045 | 1973.7 | 11.1 | 1961.6 | 1962.2 | -1.0 | 1.0 | -0.4 | 0.4 |
| M | Scand? | 18 | 1977.1 | 12.6 | 1.1284 | 0.0033 | 1993.6 | 16.5 | 1981.5 | 1982.1 | 4.4 | 4.4 | 5.0 | 5.0 |
| M | MT/WA | 18 | 1984.5 | 12.6 | 1.1242 | 0.0060 | 1994.3 | 9.8 | 1982.2 | 1982.8 | -2.3 | 2.3 | -1.7 | 1.7 |
| M | TX | 18 | 1965.3 | 12.6 | 1.3799 | 0.0038 | 1975.4 | 10.1 | 1963.3 | 1963.9 | -2.0 | 2.0 | -1.4 | 1.4 |
| M | Chile | 28 | 1967.8 | 12.6 | 1.3236 | 0.0317 | 1978.2 | 10.4 | 1966.1 | 1966.7 | -1.7 | 1.7 | -1.1 | 1.1 |
| M | Morocco | 28 | 1958.3 | 12.6 | 1.4386 | 0.0049 | 1973.4 | 15.1 | 1961.3 | 1961.9 | 3.0 | 3.0 | 3.6 | 3.6 |
| M |  | 28 | 1967.3 | 12.6 | 1.3210 | 0.0050 | 1978.4 | 11.1 | 1966.3 | 1966.9 | -1.0 | 1.0 | -0.4 | 0.4 |
| M | Scand? | 28 | 1985.7 | 12.6 | 1.1137 | 0.0031 | 1996.1 | 10.4 | 1984.0 | 1984.6 | -1.7 | 1.7 | -1.1 | 1.1 |
| M | Scand? | 28 | 1956.7 | 12.6 | 1.5728 | 0.0050 | 1968.6 | 11.9 | 1956.5 | 1957.1 | -0.2 | 0.2 | 0.4 | 0.4 |
| M | Scand? | 28 | 1949.4 | 12.6 | 1.3612 | 0.0066 | 1962.4 | 13.0 | 1950.3 | 1950.9 | 0.9 | 0.9 | 1.5 | 1.5 |
| M | CA/WA | 28 | 1980.2 | 12.6 | 1.1462 | 0.0040 | 1991.6 | 11.4 | 1979.5 | 1980.1 | -0.7 | 0.7 | -0.1 | 0.1 |
| M | MT/WA | 28 | 1984.5 | 12.6 | 1.1118 | 0.0039 | 1996.5 | 12.0 | 1984.4 | 1985.0 | -0.1 | 0.1 | 0.5 | 0.5 |
| F | Scand? | 28 | 1956.8 | 11.2 | 1.7039 | 0.0064 | 1966.4 | 9.6 | 1954.3 | 1954.9 | -2.5 | 2.5 | -1.9 | 1.9 |
| F | Scand? | 28 | 1969.3 | 11.2 | 1.3044 | 0.0044 | 1979.2 | 9.9 | 1967.1 | 1967.7 | -2.2 | 2.2 | -1.6 | 1.6 |
| F | Scand? | 28 | 1968.8 | 11.2 | 1.2973 | 0.0047 | 1979.6 | 10.8 | 1967.5 | 1968.1 | -1.3 | 1.3 | -0.7 | 0.7 |
| F | Scand? | 28 | 1977.2 | 11.2 | 1.1757 | 0.0039 | 1988.0 | 10.8 | 1975.9 | 1976.5 | -1.3 | 1.3 | -0.7 | 0.7 |
| F | Uruguay | 28 | 1985.6 | 11.2 | 1.1198 | 0.0032 | 1995.1 | 9.5 | 1983.0 | 1983.6 | -2.6 | 2.6 | -2.0 | 2.0 |
| F | Lebanon | 28 | 1985.1 | 11.2 | 1.1084 | 0.0051 | 1996.9 | 11.8 | 1984.8 | 1985.4 | -0.3 | 0.3 | 0.3 | 0.3 |
|  |  |  | **Mean** | **12.1** |  |  | **Mean** | **11.5** |  | **Mean** | **-0.6** | **1.6** | **0.0** | **1.4** |
|  |  |  |  |  |  |  | **SD** | **1.9** |  | **SD** | **1.9** | **1.1** | **1.9** | **1.2** |
|  |  |  |  |  |  |  |  |  |  |  |  |  |  |  |
| M | Scand? | 31 | 1958.2 | 2.5 | 1.2077 | 0.0047 | 1960.4 | 2.2 | 1957.9 | 1958.5 | -0.3 | 0.3 | 0.3 | 0.3 |
| M | TX | 31 | 1960.1 | 2.5 | 1.2791 | 0.0073 | 1962.0 | 1.9 | 1959.5 | 1960.1 | -0.6 | 0.6 | 0.0 | 0.0 |
| M | BC | 41 | 1962.8 | 2.5 | 1.5410 | 0.0067 | 1963.1 | 0.3 | 1960.6 | 1961.2 | -2.2 | 2.2 | -1.6 | 1.6 |
| M | TX | 41 | 1960.1 | 2.5 | 1.4131 | 0.0059 | 1962.6 | 2.5 | 1960.1 | 1960.7 | 0.0 | 0.0 | 0.6 | 0.6 |
| F | Scand? | 41 | 1970.2 | 2.5 | 1.4480 | 0.0060 | 1973.0 | 2.8 | 1970.5 | 1971.1 | 0.3 | 0.3 | 0.9 | 0.9 |
| F | Scand? | 41 | 1990.5 | 2.5 | 1.1340 | 0.0050 | 1992.8 | 2.3 | 1990.3 | 1990.9 | -0.2 | 0.2 | 0.4 | 0.4 |
| M |  | 41 | 1973.0 | 2.5 | 1.3982 | 0.0052 | 1974.6 | 1.6 | 1972.1 | 1972.7 | -0.9 | 0.9 | -0.3 | 0.3 |
|  |  |  | **Mean** | **2.5** |  |  | **Mean** | **1.9** |  | **Mean** | **-0.6** | **0.6** | **0.0** | **0.6** |
|  |  |  |  |  |  |  | **SD** | **0.8** |  | **SD** | **0.8** | **0.7** | **0.8** | **0.5** |
|  |  |  |  |  |  |  |  |  |  |  |  |  |  |  |
| M | TX | 32 | 1960.1 | 3.0 | 1.6571 | 0.1693 | 1963.3 | 3.2 | 1960.3 | 1960.1 | 0.2 | 0.2 | 0.0 | 0.0 |
| M | Scand? | 42 | 1960.6 | 3.0 | 1.3830 | 0.0070 | 1962.5 | 1.9 | 1959.5 | 1959.3 | -1.1 | 1.1 | -1.3 | 1.3 |
| M | TX | 42 | 1960.1 | 3.0 | 1.5051 | 0.1504 | 1962.8 | 2.7 | 1959.8 | 1959.6 | -0.3 | 0.3 | -0.5 | 0.5 |
| M | Scand? | 42 | 1973.0 | 3.0 | 1.3951 | 0.0052 | 1974.7 | 1.7 | 1971.7 | 1971.5 | -1.3 | 1.3 | -1.5 | 1.5 |
| M | Sweden | 42 | 1989.1 | 3.0 | 1.1481 | 0.0043 | 1991.4 | 2.3 | 1988.4 | 1988.2 | -0.7 | 0.7 | -0.9 | 0.9 |
| F | Sweden | 42 | 1958.1 | 2.8 | 1.6568 | 0.0062 | 1966.8 | 8.7 | 1963.8 | 1963.6 | 5.7 | 5.7 | 5.5 | 5.5 |
| M | Georgia | 42 | 1981.1 | 3.0 | 1.2294 | 0.0046 | 1983.3 | 2.2 | 1980.3 | 1980.1 | -0.8 | 0.8 | -1.0 | 1.0 |
|  |  |  | **Mean** | **3.0** |  |  | **Mean** | **3.2** |  | **Mean** | **0.2** | **1.4** | **0.0** | **1.5** |
|  |  |  |  |  |  |  | **SD** | **2.5** |  | **SD** | **2.5** | **1.9** | **2.5** | **1.8** |
|  |  |  |  |  |  |  |  |  |  |  |  |  |  |  |
| M | n/a | 33 | 1962.6 | 4.3 | 1.5190 | 0.0060 | 1967.1 | 4.5 | 1962.8 | 1963.5 | 0.2 | 0.2 | 0.9 | 0.9 |
| M | TX | 43 | 1960.1 | 4.3 | 1.4475 | 0.0062 | 1962.8 | 2.7 | 1958.5 | 1959.2 | -1.6 | 1.6 | -0.9 | 0.9 |
| M | Bosnia | 33 | 1953.0 | 4.3 | 1.0126 | 0.0039 | 1956.1 | 3.1 | 1951.8 | 1952.5 | -1.2 | 1.2 | -0.5 | 0.5 |
| M | Bosnia | 43 | 1953.0 | 4.3 | 1.0634 | 0.0507 | 1957.2 | 4.2 | 1952.9 | 1953.6 | -0.1 | 0.1 | 0.6 | 0.6 |
|  |  |  | **Mean** | **4.3** |  |  | **Mean** | **3.6** |  | **Mean** | **-0.7** | **0.8** | **0.0** | **0.7** |
|  |  |  |  |  |  |  | **SD** | **0.9** |  | **SD** | **0.9** | **0.7** | **0.9** | **0.2** |
|  |  |  |  |  |  |  |  |  |  |  |  |  |  |  |
| M | Sweden | 34 | 1991.8 | 5.1 | 1.1145 | 0.0036 | 1996.0 | 4.2 | 1991.1 | 1991.9 | -0.7 | 0.7 | 0.1 | 0.1 |
| M | Sweden | 34 | 1988.6 | 5.1 | 1.1366 | 0.0076 | 1992.7 | 4.1 | 1987.8 | 1988.6 | -0.8 | 0.8 | 0.0 | 0.0 |
| M | Scand? | 34 | 1990.0 | 5.1 | 1.1080 | 0.0040 | 1997.2 | 7.2 | 1992.3 | 1993.1 | 2.3 | 2.3 | 3.1 | 3.1 |
| M | Sweden | 34 | 1990.8 | 5.1 | 1.1212 | 0.0043 | 1995.0 | 4.2 | 1990.1 | 1990.9 | -0.7 | 0.7 | 0.1 | 0.1 |
| M | CT | 34 | 1996.3 | 5.1 | 1.0872 | 0.0038 | 2001.0 | 4.7 | 1996.1 | 1996.9 | -0.2 | 0.2 | 0.6 | 0.6 |
| F | Sweden | 34 | 1993.6 | 4.4 | 1.1008 | 0.0039 | 1998.4 | 4.8 | 1993.5 | 1994.3 | -0.1 | 0.1 | 0.7 | 0.7 |
| F | Sweden | 34 | 1991.4 | 4.4 | 1.1196 | 0.0037 | 1995.2 | 3.8 | 1990.3 | 1991.1 | -1.1 | 1.1 | -0.3 | 0.3 |
| F | TX | 34 | 1991.4 | 4.4 | 1.1224 | 0.0032 | 1994.6 | 3.2 | 1989.7 | 1990.5 | -1.7 | 1.7 | -0.9 | 0.9 |
| F | Sweden | 44 | 1988.8 | 4.4 | 1.1381 | 0.0033 | 1992.2 | 3.4 | 1987.3 | 1988.1 | -1.5 | 1.5 | -0.7 | 0.7 |
| F | Sweden | 44 | 1992.5 | 4.4 | 1.1156 | 0.0042 | 1995.8 | 3.3 | 1990.9 | 1991.7 | -1.6 | 1.6 | -0.8 | 0.8 |
| M | Sweden | 44 | 1991.3 | 5.1 | 1.1218 | 0.0049 | 1995.0 | 3.7 | 1990.1 | 1990.9 | -1.2 | 1.2 | -0.4 | 0.4 |
| M | Sweden | 44 | 1993.2 | 5.1 | 1.1054 | 0.0035 | 1997.7 | 4.5 | 1992.8 | 1993.6 | -0.4 | 0.4 | 0.4 | 0.4 |
| M | BC | 44 | 1996.6 | 5.1 | 1.1130 | 0.0042 | 1996.3 | -0.3 | 1991.4 | 1992.2 | -5.2 | 5.2 | -4.4 | 4.4 |
| M | CT | 44 | 1997.2 | 5.1 | 1.0940 | 0.0039 | 2000.0 | 2.8 | 1995.1 | 1995.9 | -2.1 | 2.1 | -1.3 | 1.3 |
| M | TX | 44 | 1960.1 | 5.1 | 1.6666 | 0.0067 | 1966.4 | 6.3 | 1961.5 | 1962.3 | 1.4 | 1.4 | 2.2 | 2.2 |
| M | Scand? | 44 | 1962.6 | 5.1 | 1.5840 | 0.0070 | 1968.3 | 5.7 | 1963.4 | 1964.2 | 0.8 | 0.8 | 1.6 | 1.6 |
|  |  |  | **Mean** | **4.9** |  |  | **Mean** | **4.1** |  | **Mean** | **-0.8** | **1.4** | **0.0** | **1.1** |
|  |  |  |  |  |  |  | **SD** | **1.7** |  | **SD** | **1.7** | **1.2** | **1.7** | **1.2** |
|  |  |  |  |  |  |  |  |  |  |  |  |  |  |  |
| F | Sweden | 35 | 1989.2 | 5.7 | 1.1211 | 0.0037 | 1994.9 | 5.7 | 1988.9 | 1989.6 | -0.3 | 0.3 | 0.4 | 0.4 |
| M | Uruguay | 35 | 1966.8 | 6.5 | 1.4720 | 0.0043 | 1972.4 | 5.6 | 1966.4 | 1967.1 | -0.4 | 0.4 | 0.3 | 0.3 |
| F | Scand? | 45 | 1973.7 | 5.7 | 1.3231 | 0.0044 | 1978.4 | 4.7 | 1972.4 | 1973.1 | -1.3 | 1.3 | -0.6 | 0.6 |
| F | Mexico | 45 | 1963.8 | 5.7 | 1.5331 | 0.0058 | 1970.2 | 6.4 | 1964.2 | 1964.9 | 0.4 | 0.4 | 1.1 | 1.1 |
| F | WA | 45 | 1962.8 | 5.7 | 1.6356 | 0.0057 | 1967.4 | 4.6 | 1961.4 | 1962.1 | -1.4 | 1.4 | -0.7 | 0.7 |
| M | Sweden | 45 | 1992.2 | 6.5 | 1.1020 | 0.0039 | 1998.2 | 6.0 | 1992.2 | 1992.9 | 0.0 | 0.0 | 0.7 | 0.7 |
| M | MA | 45 | 1957.7 | 6.5 | 1.6698 | 0.0072 | 1962.0 | 4.3 | 1956.0 | 1956.7 | -1.7 | 1.7 | -1.0 | 1.0 |
|  |  |  | **Mean** | **6.0** |  |  | **Mean** | **5.3** |  | **Mean** | **-0.7** | **0.8** | **0.0** | **0.7** |
|  |  |  |  |  |  |  | **SD** | **0.8** |  | **SD** | **0.8** | **0.7** | **0.8** | **0.3** |
|  |  |  |  |  |  |  |  |  |  |  |  |  |  |  |
| M | Chile | 36 | 1967.8 | 2.4 | 1.5222 | 0.0118 | 1970.8 | 3.0 | 1968.4 | 1967.8 | 0.6 | 0.6 | 0.0 | 0.0 |
| M | Scand? | 36 | 1955.8 | 2.4 | 0.9934 | 0.0031 | 1955.1 | -0.7 | 1952.7 | 1952.1 | -3.1 | 3.1 | -3.7 | 3.7 |
| M | Mexico | 36 | 1954.8 | 2.4 | 1.0738 | 0.0038 | 1957.4 | 2.6 | 1955.0 | 1954.4 | 0.2 | 0.2 | -0.4 | 0.4 |
| M | TX | 36 | 1992.0 | 2.4 | 1.1019 | 0.0035 | 1998.2 | 6.2 | 1995.8 | 1995.2 | 3.8 | 3.8 | 3.2 | 3.2 |
| F | Uruguay | 36 | 1992.3 | 2.3 | 1.1158 | 0.0036 | 1995.7 | 3.4 | 1993.3 | 1992.7 | 1.0 | 1.0 | 0.4 | 0.4 |
| F | Na | 36 | 1963.5 | 2.3 | 1.6320 | 0.0070 | 1967.4 | 3.9 | 1965.0 | 1964.4 | 1.5 | 1.5 | 0.9 | 0.9 |
| M | TX | 46 | 1965.3 | 2.4 | 1.4075 | 0.0041 | 1962.6 | -2.7 | 1960.2 | 1959.6 | -5.1 | 5.1 | -5.7 | 5.7 |
| F | BC | 36 | 1970.4 | 2.3 | 1.3631 | 0.0058 | 1976.6 | 6.2 | 1974.2 | 1973.6 | 3.8 | 3.8 | 3.2 | 3.2 |
| F | Sweden | 46 | 1976.6 | 2.3 | 1.2552 | 0.0054 | 1981.6 | 5.0 | 1979.2 | 1978.6 | 2.6 | 2.6 | 2.0 | 2.0 |
|  |  |  | **Mean** | **2.4** |  |  | **Mean** | **3.0** |  | **Mean** | **0.6** | **2.4** | **0.0** | **2.2** |
|  |  |  |  |  |  |  | **SD** | **3.0** |  | **SD** | **3.0** | **1.7** | **3.0** | **1.9** |
|  |  |  |  |  |  |  |  |  |  |  |  |  |  |  |
| M | Scand? | 37 | 1979.4 | 6.5 | 1.2200 | 0.0047 | 1983.9 | 4.5 | 1977.7 | 1977.7 | -1.7 | 1.7 | -1.7 | 1.7 |
| M | Mexico | 37 | 1954.8 | 6.5 | 1.6573 | 0.0059 | 1962.7 | 7.9 | 1956.5 | 1956.5 | 1.7 | 1.7 | 1.7 | 1.7 |
| M | TX | 37 | 1960.1 | 6.5 | 1.6735 | 0.0056 | 1966.8 | 6.7 | 1960.6 | 1960.6 | 0.5 | 0.5 | 0.5 | 0.5 |
| M | WA | 37 | 1970.6 | 6.5 | 1.3518 | 0.0048 | 1976.8 | 6.2 | 1970.6 | 1970.6 | 0.0 | 0.0 | 0.0 | 0.0 |
| M | Scand? | 37 | 1971.7 | 6.5 | 1.2710 | 0.0050 | 1980.8 | 9.1 | 1974.6 | 1974.6 | 2.9 | 2.9 | 2.9 | 2.9 |
| F | Scand? | 37 | 1963.7 | 5.6 | 1.4810 | 0.0060 | 1972.0 | 8.3 | 1965.8 | 1965.8 | 2.1 | 2.1 | 2.1 | 2.1 |
| F | Sweden | 47 | 1963.7 | 5.6 | 1.5644 | 0.0057 | 1969.6 | 5.9 | 1963.4 | 1963.4 | -0.3 | 0.3 | -0.3 | 0.3 |
| F | Scand? | 47 | 1977.8 | 5.6 | 1.2320 | 0.0050 | 1983.2 | 5.4 | 1977.0 | 1977.0 | -0.8 | 0.8 | -0.8 | -0.8 |
| M | Scand? | 47 | 1962.4 | 6.5 | 1.6172 | 0.0060 | 1967.6 | 5.2 | 1961.4 | 1961.4 | -1.0 | 1.0 | -1.0 | 1.0 |
| M | TX | 47 | 1960.1 | 6.5 | 1.6945 | 0.0060 | 1966.5 | 6.4 | 1960.3 | 1960.3 | 0.2 | 0.2 | 0.2 | 0.2 |
| M | WA | 47 | 1973.4 | 6.5 | 1.3421 | 0.0047 | 1977.5 | 4.1 | 1971.3 | 1971.3 | -2.1 | 2.1 | -2.1 | 2.1 |
| F | Irak | 47 | 1967.6 | 5.6 | 1.4720 | 0.0055 | 1972.3 | 4.7 | 1966.1 | 1966.1 | -1.5 | 1.5 | -1.5 | 1.5 |
|  |  |  | **Mean** | **6.2** |  |  | **Mean** | **6.2** |  | **Mean** | **0.0** | **1.2** | **0.0** | **1.1** |
|  |  |  |  |  |  |  | **SD** | **1.6** |  | **SD** | **1.6** | **0.9** | **1.6** | **1.1** |
|  |  |  |  |  |  |  |  |  |  |  |  |  |  |  |
| M | Marocko | 38 | 1958.3 | 13.0 | 1.5362 | 0.0055 | 1970.0 | 11.7 | 1957.4 | 1959.0 | -0.9 | 0.9 | 0.7 | 0.7 |
| M | Scand? | 38 | 1950.6 | 13.0 | 1.1802 | 0.0031 | 1957.8 | 7.2 | 1945.2 | 1946.8 | -5.4 | 5.4 | -3.8 | 3.8 |
| M | WA | 38 | 1982.3 | 13.0 | 1.1357 | 0.0040 | 1992.5 | 10.2 | 1979.9 | 1981.5 | -2.4 | 2.4 | -0.8 | 0.8 |
| F | Scand? | 38 | 1971.2 | 11.8 | 1.2452 | 0.0040 | 1982.4 | 11.2 | 1969.8 | 1971.4 | -1.4 | 1.4 | 0.2 | 0.2 |
| F | Chile | 38 | 1955.9 | 11.8 | 1.5473 | 0.0054 | 1970.0 | 14.1 | 1957.4 | 1959.0 | 1.5 | 1.5 | 3.1 | 3.1 |
| M | Quwait | 48 | 1970.4 | 13.0 | 1.2715 | 0.0050 | 1981.0 | 10.6 | 1968.4 | 1970.0 | -2.0 | 2.0 | -0.4 | 0.4 |
| M | Scand? | 48 | 1957.4 | 13.0 | 1.5586 | 0.0063 | 1969.3 | 11.9 | 1956.7 | 1958.3 | -0.7 | 0.7 | 0.9 | 0.9 |
| M | Scand? | 48 | 1964.9 | 13.0 | 1.3236 | 0.0040 | 1978.3 | 13.4 | 1965.7 | 1967.3 | 0.8 | 0.8 | 2.4 | 2.4 |
| M | WA | 48 | 1982.3 | 13.0 | 1.1323 | 0.0042 | 1993.1 | 10.8 | 1980.5 | 1982.1 | -1.8 | 1.8 | -0.2 | 0.2 |
| F | Scand? | 48 | 1961.7 | 11.8 | 1.5186 | 0.0063 | 1970.9 | 9.2 | 1958.3 | 1959.9 | -3.4 | 3.4 | -1.8 | 1.8 |
|  |  |  | **Mean** | **12.6** |  |  | **Mean** | **11.0** |  | **Mean** | **-0.8** | **1.5** | **0.0** | **1.1** |
|  |  |  |  |  |  |  | **SD** | **1.9** |  | **SD** | **1.7** | **1.1** | **1.5** | **1.0** |

^1^In the four studies, teeth were collected by dentists in different countries. Information about country of origin was not recorded by Swedish dentists in the first publication (6), but n/a typically implies that the subject either was raised in Europe or Middle East. Scand? = probably raised in Scandinavia.

^2^Enamel formation time according to Nolla [20].

^3^DOB of the person was calculated by subtracting the actual DOB from the average estimated ^14^C incorporation time. When doing so, the average error mathematically becomes 0.0 in the second last column.

^4^Unclear when this subject moved to WA.
