# Supplementary material for: Analysis of Radiocarbon, Stable Isotopes and DNA in Teeth to Facilitate Identification of Unknown Decedents
Source: PLoS One. 2013 Jul 29;8(7):e69597. doi: 10.1371/journal.pone.0069597 (PMC3726681; doi:10.1371/journal.pone.0069597)
Supplement: Supporting Information S1 — Supporting file containing Tables S3–S5. Table S3. Mean difference between 14C-measured age and Nolla's calibration. Positive values indicate that the measured age at tooth formation is larger than Nolla's estimate. Most values are negative, indicating that the incorporation of radiocarbon on average is taking place earlier than the enamel radio-opacity according to Nolla's stage 4.5 estimate. Table S4. Standard deviation in age at tooth formation for each tooth type and jaw. Table S5. Confidence intervals for the date of birth given the enamel formation year. Note that this does not use Nolla's estimates, only the 14C calibrated values. (DOC) [file pone.0069597.s003.doc]

**Supporting information**

**1. Detailed analytical data**

Table S1 shows detailed data supporting Table 2. The ^14^C/C ratios are corrected for isotope fractionation and expressed in both fraction modern, F^14^C , and decay corrected Δ^14^C nomenclatures. Whenever δ^13^C could not be analyzed due to small sample size, the δ^13^C was estimated from the average of teeth from that region with an uncertainty of ± 2‰. Table S1 includes several teeth from some subjects. However, since the intra-individual results show similar variation as the inter-individual variation, their inclusion will not affect the overall error much.

Table S2 constitutes a compilation of the ^14^C birth dating of tooth enamel included in this publication as well as such data from the three previous studies published by our laboratory . Only teeth prepared using the standard procedure – immersion in concentrated NaOH during sonication – are included. Cryo-cracking of tooth crowns and dissolution of whole crown are more rapid techniques, and might give similar precision. However, since the number of samples prepared by these methods is limited, we only present results from samples, in which the enamel has been isolated by the standard procedure. The table compares the precision of ^14^C DOB estimation using reference information on enamel formation time with DOB estimation using the average ^14^C incorporation time. It should be kept in mind that the ^14^C levels in tooth enamel is not exactly the same as the contemporary atmospheric levels since there is a lag between atmosphere and human as carbon moves up the food chain . Therefore the ^14^C incorporation times are not an exact indicator of enamel formation time.

**References**

1. Reimer PJ, Brown TA, Reimer RW (2004) Discussion: Reporting and calibration of post-bomb C-14 data. Radiocarbon 46: 1299-1304.

2. Stuiver M, Polach, H. A. (1977) Discussion: reporting of 14C data. Radiocarbon 19: 355-363.

3. Alkass K, Buchholz BA, Druid H, Spalding KL (2011) Analysis of 14C and 13C in teeth provides precise birth dating and clues to geographical origin. Forensic Sci Int 209: 34-41.

4. Alkass K, Buchholz BA, Ohtani S, Yamamoto T, Druid H, et al. (2010) Age estimation in forensic sciences: application of combined aspartic acid racemization and radiocarbon analysis. Molecular & Cellular Proteomics 9: 1022-1030.

5. Spalding KL, Buchholz BA, Bergman LE, Druid H, Frisen J (2005) Forensics: age written in teeth by nuclear tests. Nature 437: 333-334.

6. Nolla CM (1960) The development of the permanent teeth. J Dental Child 27.

7. Libby WF, Berger R, Mead JF, Alexander GV, Ross JF (1964) Replacement Rates for Human Tissue from Atmospheric Radiocarbon. Science 146: 1170-1172.

8. Nydal R, Lovseth K (1965) Distribution of Radiocarbon from Nuclear Tests. Nature 206: 1029-1031.

**2. Error calculations for the date of birth of person**

^14^C abundance values are available from different types of teeth from a large cohort of subjects (n=165) born between 1949 and 1997. Tooth samples come from canines, central incisors, first molars, first premolars, lateral incisors, second molars, second premolars, or third molars, either from the maxillary or mandibular jaw. Teeth are formed after birth at specific ages, so by dating precisely the formation date of the tooth, it is possible to infer the date of birth of the person.

Nolla (1960) compiled the age at 9 different stages of tooth formation, with the formation of enamel occurring during stages 4-5. ^14^C content in the enamel should then correspond to the time of stage 4.5. Since the birth date of each subject is known, it is possible to compare the prediction of time of tooth formation (according to Nolla's calibration) and the time of formation according to the ^14^C measurement.

***14C dating method***

Enamel formation occurs in a relatively short period of time and it is assumed that enamel is not replaced after initial formation. ^14^C content in enamel should therefore reflect atmospheric^14^C levels at time of formation. This allows a direct birth-dating of the tooth, by finding the year for which the atmospheric level coincides with the measured enamel ^14^C level.

This method poses two problems. First, for subjects born before 1963, two dates correspond to ^14^C levels, one during the rise of atmospheric ^14^C and one during the decline. For most subjects, one can distinguish which date to pick, but for subject born just before 1963, the two possible birth dates are close to each other. The second problem is that of the bomb curve smoothing: the bomb curve should be smoothed appropriately before dating the tooth, since the time span for carbon incorporation into the food chain and into the enamel is probably of the order of 0.5-1 year.

The online resource CALIBOMB is useful to date individual samples, but here we used a smoothed (over a year) bomb curve and the R software to automatically date all samples. Two birth date estimates were obtained for each sample. If the subject was born after 1963, the uphill date was chosen. If the subject was born before 1963, the uphill and downhill estimates were compared with Nolla's table and the date closest to Nolla's was chosen.. This method does not guarantee that this is the actual birth date of the tooth, but we refer to it as the “best” estimate.

***Results and discussion***

In order to compare the ^14^C -measured birth date with the one obtained using Nolla's reference enamel formation time, we looked at the age of the subject at the time of the tooth formation by subtracting the subject birth date from the tooth formation date, and compared the resulting difference with crude ^14^C results. ^14^C -measured and Nolla's date of tooth formation correlated well, with R=0.88, (p-value < 1e-15). The mean difference (in years) by tooth type and jaw is given in Table S3. This table shows that mandibular tooth measurements (mean difference -0.26 years) are closer to Nolla's than maxillary tooth measurements (mean difference -0.86 years). Apart from first molars, molars and premolars formation age estimates are within few months from Nolla's estimates.

The systematic deviation seen between Nolla's and ^14^C calibrations may be due to several factors. Perhaps ^14^C is incorporated before stage 4.5. In such case, an earlier stage should be selected for calibration. Another possible bias is the delay between ^14^C incorporation in the food chain and the incorporation into the tooth. However, if the carbon incorporated in the tooth is in average X years old, we would have to remove X years from the tooth formation date. This would induce a much larger difference between the data and Nolla's, so this is unlikely to account for much of the bias. Independently from these considerations, as long as the bias is systematic, it is possible to construct a calibration table to estimate the birth date of an individual based on the formation date of the tooth.

**Table S3**. Mean difference between ^14^C-measured age and Nolla's calibration. Positive values indicate that the measured age at tooth formation is larger than Nolla's estimate. Most values are negative, indicating that the incorporation of radiocarbon on average is taking place earlier than the enamel radio-opacity according to Nolla's stage 4.5 estimate.

|  | central incisors | lateral incisor | canine | first premolar | second premolar | first molar | second molar | third molar |
| --- | --- | --- | --- | --- | --- | --- | --- | --- |
| Maxillary | -1.27 | -0.08 | -0.90 | -1.09 | -1.07 | -1.48 | -0.83 | -0.19 |
| Mandibular | -0.56 | -0.19 | -1.75 | -0.18 | -0.15 | 2.11 | 0.05 | -1.40 |

There is considerable formation date variability between samples. Identifying the magnitude of variability is crucial for assessing birth date accuracy. Table S4 shows the variability in dating each tooth type and jaw, in standard deviation (years). The SD is in average 1.40 years, giving a rough estimate on the precision of the date of birth of the subject, by subtracting the mean formation time from the measured tooth formation date. If the formation age is normally distributed, we would expect a 95% confidence interval of mean ± 1.96*SD, i.e. an error of ± 2.74 years. Of course, for a given tooth type, the confidence interval might be smaller of larger, depending on the standard deviation for that type.

**Table S4**. Standard deviation in age at tooth formation for each tooth type and jaw.

|  | central incisors | lateral incisor | canine | first premolar | second premolar | first molar | second molar | third molar |
| --- | --- | --- | --- | --- | --- | --- | --- | --- |
| Maxillary | 1.68 | 1.32 | 1.51 | 1.35 | 1.58 | 0.98 | 0.79 | 1.86 |
| Mandibular | 1.24 | 1.20 | 1.61 | 1.48 | 0.90 | 1.61 | 1.61 | 1.66 |

To make the confidence interval estimate more precise, we ran a bootstrap test by resampling the deviation between the age at tooth formation and the mean of the age at tooth formation for each tooth type and jaw. The number of bootstrap replicates was R=10000, and the 95%, 90%, and 67% confidence intervals were calculated. The confidence intervals are shown in Table S5.

**Table S5.** Confidence intervals for the date of birth given the enamel formation year. Note that this does not use Nolla's estimates, only the ^14^C calibrated values.

| CI | Lower Bound (years) | Upper Bound (year) |
| --- | --- | --- |
| 95% | -2.32 | 2.73 |
| 90% | -2.03 | 2.42 |
| 67% | -1.31 | 1.34 |

Given a tooth formation year, we can predict that the birth date will fall within a range of 5 years (2.32 + 2.73 = 5.05 years) with a probability of 95%. This is a more optimistic confidence interval than computed assuming a normal distribution of deviation, which is almost 6 years in range.

The bootstrap was applied on all tooth samples, regardless of the type and jaw. However, Table S3 shows that some type/jaw might provide a better estimate. For instance, maxillary second molar show a SD of only 0.79 years (n=11). Applying the bootstrap method to maxillary second molar only yields a 95% confidence interval of [-0.92, 1.16], i.e. a range of slightly more than 2 years. The normality assumption would give a 95% CI estimate of 2*1.96*0.79 = 3.1 years.
